# Cell Granularity Reflects Immune Cell Function and Enables Selection of Lymphocytes with Superior Attributes for Immunotherapy

**DOI:** 10.1002/advs.202302175

**Published:** 2023-08-06

**Authors:** Tongjin Wu, Joel Heng Loong Tan, Wei‐Xiang Sin, Yen Hoon Luah, Sue Yee Tan, Myra Goh, Michael E. Birnbaum, Qingfeng Chen, Lih Feng Cheow

**Affiliations:** ^1^ Department of Biomedical Engineering Faculty of Engineering National University of Singapore Singapore 117583 Singapore; ^2^ Institute for Health Innovation and Technology National University of Singapore Singapore 117599 Singapore; ^3^ Institute of Molecular and Cell Biology (IMCB) Agency for Science Technology and Research (A*STAR) Singapore 138673 Singapore; ^4^ Critical Analytics for Manufacturing of Personalized Medicine Singapore‐MIT Alliance for Research and Technology Singapore 138602 Singapore; ^5^ Department of Biological Engineering Massachusetts Institute of Technology Cambridge MA 02139 USA

**Keywords:** cell granularity, immunotherapy, lymphocyte, side scatter, T cells

## Abstract

In keeping with the rule of “form follows function”, morphological aspects of a cell can reflect its role. Here, it is shown that the cellular granularity of a lymphocyte, represented by its intrinsic side scatter (SSC), is a potent indicator of its cell state and function. The granularity of a lymphocyte increases from naïve to terminal effector state. High‐throughput cell‐sorting yields a SSC^high^ population that can mediate immediate effector functions, and a highly prolific SSC^low^ population that can give rise to the replenishment of the memory pool. CAR‐T cells derived from the younger SSC^low^ population possess desirable attributes for immunotherapy, manifested by increased naïve‐like cells and stem cell memory (T_SCM_)‐like cells together with a balanced CD4/CD8 ratio, as well as enhanced target‐killing in vitro and in vivo. Altogether, lymphocyte segregation based on biophysical properties is an effective approach for label‐free selection of cells that share collective functions and can have important applications for cell‐based immunotherapies.

## Graphical abstract

Cell granularity correlates with its state and function. T‐cell subsets of younger phenotypes and functionality have a lower granularity. This biophysical property enables the efficient enrichment of these favorable T cells through their side scattering (SSC) properties. Isolated T‐cell populations show promises for improved immunotherapy associated with optimized manufacturing procedures and therapeutic efficacy.

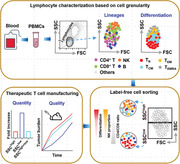

## 1 Introduction

The functional heterogeneity of immune cells is a topic of great fascination to immunologists. Throughout the years, immunologists have had great success in correlating various cellular phenotypes (e.g., differentiation status, function) with cell surface marker expression.^[^ ^1^, ^2^, ^3^ ^]^ However, surface proteins only account for a fraction of total proteins in cells,^[^ ^4^ ^]^ the exact functional roles of many cell surface markers are still unknown, and many cell attributes may not correlate with the presence or expression level of individual cell surface proteins.

While it can be challenging to profile specific intracellular functional states in live cells, many functional changes in cells can lead to stereotypical changes in cellular morphology.^[^ ^5^ ^]^ Biophysical properties of a cell, including its size, shape, and distribution of intracellular structures can therefore represent emergent properties of cell phenotype that can inform of its physiological and signaling states.^[^ ^6^, ^7^ ^]^ The cell membrane and cytoplasm are mostly transparent due to their low light absorption and scattering properties, but organelles within the cells such as the nucleus, granules, and mitochondria can scatter light effectively making them distinguishable under visible light.^[^ ^7^ ^]^ Using instruments that can measure comprehensive light scattering profiles (LSP), it has been demonstrated that the main lymphocyte populations such as T cells versus B cells,^[^ ^8^ ^]^ CD4^+^ T versus CD8^+^ T cells,^[^ ^9^ ^]^ as well as CD56^bright^ NK versus CD56^dim^ NK subclasses^[^ ^10^ ^]^ can be distinguished.

While these reports support the basis of using light scattering information to distinguish lymphocyte subpopulations, the small angle light scattering^[^ ^9^, ^10^ ^]^ and scanning flow cytometry^[^ ^8^ ^]^ instruments needed for performing LSP measurements are only available in very specialized laboratories, have limited throughputs (<1000 cells analyzed per hour), and do not have the capability to sort cells. In contrast, flow cytometers are widely available, can sort cells at high throughput (>10 000 cells s^−1^), and can provide fixed‐angled measurements of light scattering. In flow cytometry, the front scatter (FSC, along the path of the laser beam) measurement correlates to cell size while the side scatter (SSC, perpendicular to the laser beam) measurement provides robust information about the internal complexity and granularity of a cell. In fact, SSC measurement is the key to five‐part white blood cell differential measurements (distinguishing neutrophils, eosinophils, basophils, lymphocytes, and monocytes) in automated hematology analysis that has existed since the 1970s and is still widely used today.^[^ ^11^, ^12^ ^]^ Nonetheless, higher resolution characterization and enrichment of lymphocyte subpopulations, especially for T cells of different differentiation states (e.g., naïve, memory, and effector), based on SSC from conventional flow cytometry have not been reported.

Cytotoxic T cells and NK cells are characterized by pre‐stored granules containing cytotoxic factors such as granzymes and perforin.^[^ ^13^, ^14^ ^]^ T cell function has also been associated with the biogenesis and morphology change of mitochondria.^[^ ^15^, ^16^, ^17^ ^]^ Under a transmission electron microscope clear difference was observed between effector T cells and naive cells, as evident from their increased lytic granules and mitochondria.^[^ ^18^ ^]^ 3D refractive index tomography also showed that CD4^+^ T cells, CD8^+^ T cells, and B cells can be separated based on their optical properties.^[^ ^19^ ^]^ The ability to effectively identify lymphocytes based on their intrinsic optical properties could provide a strategy to segregate cells based on their morphological design principles. Furthermore, this could yield a novel label‐free approach for selecting particular functional lymphocyte populations in clinical applications such as immunotherapy, overcoming many fundamental challenges that arise from the use of antibodies.

**Figure 1:**
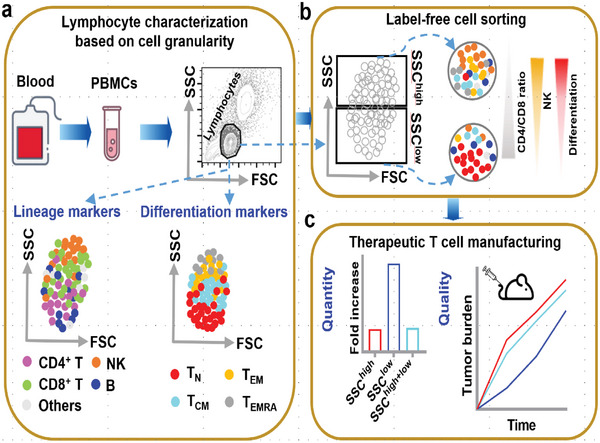
Illustration of functional immune cell state embodied in cellular granularity and its application for improving T cell manufacturing. a) Cellular subpopulations representing different phenotypic and differentiation states in human PBMC subpopulations can be distinguished by their side scattering profiles. b) Unstained lymphocytes sorted into SSC^high^ (top 50% SSC) and SSC^low^ (bottom 50% SSC) populations have distinct cell composition and functions. c) Incorporation of label‐free cell segregation based on granularity in CAR‐T manufacturing produces cellular products with superior attributes for immunotherapy.

In this manuscript, we demonstrate that the cell granularity measured by SSC in flow cytometry can be used to segregate functional lymphocyte subpopulations (**Figure** **1**a,b). CD4^+^ as well as less differentiated T cells including naïve (T_N_)^[^ ^1^ ^]^ and early central memory (T_CM_) cells were found to have low SSC, while CD8^+^, NK, and more differentiated T cells (late T_CM_, effector memory T_EM,_ and terminal effector T_EMRA_)^[^ ^2^, ^20^ ^]^ have high SSC values. Very interestingly, the SSC index ranks cells according to the anatomy of an immune response. On one end of the spectrum there are the SSC^low^ inexperienced T_N_ cells, on the other end of the spectrum there are the SSC^high^ specialized effector T cells and NK cells that contain numerous cytotoxic granules.^[^ ^14^ ^]^ The location of memory T cells in between is consistent with the expectation that they have intermediate amounts of granules^[^ ^21^ ^]^ to support their rapid response when they re‐encounter specific antigens.^[^ ^2^ ^]^ SSC^low^ lymphocytes also expand at a higher rate compared to an unsorted population suggesting a coupling of proliferation capacity with cellular functions.

Clinical^[^ ^22^, ^23^, ^24^, ^25^ ^]^ and preclinical^[^ ^26^, ^27^, ^28^, ^29^, ^30^ ^]^ studies have provided strong evidence that adoptive transfusion of younger therapeutic T cells, for example, chimeric antigen receptor (CAR)‐T cells that are derived from or containing more T_N_, stem‐cell memory (T_SCM_),^[^ ^31^ ^]^ and T_CM_, predicts better outcomes in vivo. The functional dichotomy of SSC‐sorted populations suggests potential use for optimal cell manufacturing in adoptive cell immunotherapy (Figure 1b,c). We demonstrate that CD19‐targeting CAR‐T cells prepared from SSC^low^ lymphocytes have enhanced viral transduction efficiency and transgenic stability, as well as yielding a more favorable CD4/CD8 ratio and generating effector cells that have higher markers of persistence. These CAR‐T cells are also endowed with prolonged antitumor activity in vitro against recursive stimulation of leukemia cells. Finally, in vivo mouse experiments showed enhanced initial tumor growth control by CAR‐T cells derived from SSC^low^ lymphocytes, corroborating in vitro findings, Our study proposes an original approach in the label‐free selection of favorable T cells based on optical side scattering to improve the manufacturing of adoptive immunotherapy product with long‐term efficacy (Figure 1b,c).

## 2 Results

### 2.1 Side Scatter Measurements Robustly Segregate Functional Lymphocyte Subpopulations

Modern flow cytometry instruments are capable of discerning small variations in cellular optical properties.^[^ ^32^ ^]^ Two of the basic parameters that are obtained from flow cytometry analysis are the FSC and SSC measurements. While the FSC is normally related to cell size, the SSC is associated with cellular complexity or granularity. An increased number of organelles in the cell cytoplasm will result in a higher SSC measurement. While this concept has proven to be useful for distinguishing between granulocytes,^[^ ^33^ ^]^ monocytes, and lymphocytes (e.g., cytotoxic cells versus B cells) due to their notable difference in granularity,^[^ ^8^, ^9^, ^10^, ^11^, ^34^ ^]^ there has been little effort to date to explore the subtle difference in cell granularity within the T lymphocyte subpopulation (e.g., differentiation state of T cells) using conventional high throughput flow cytometry.

**Figure 2:**
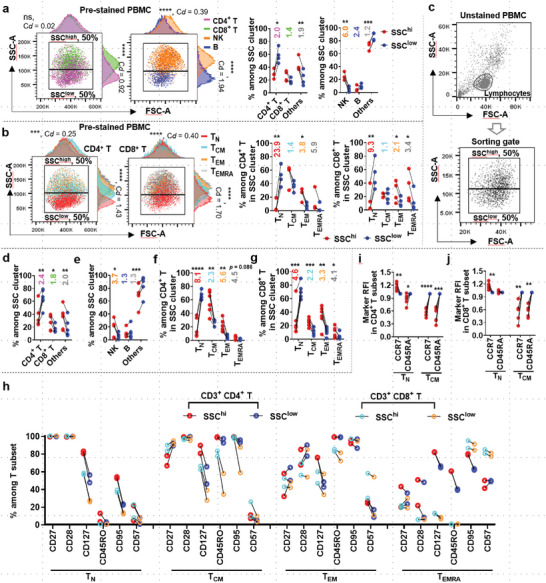
SSC is a robust biophysical property for the characterization and enrichment of functional lymphocytes. a,b) FSC/SSC distribution of CD4^+^ T/CD8^+^ T/NK/B cells (a), and T_N_/T_CM_/T_EM_/T_EMRA_ cell subsets among SSC^high^ (top 50% SSC) and SSC^low^ (bottom 50% SSC) lymphocyte clusters of pre‐stained PBMCs (b). A total of at least 20000 lymphocytes were included for analysis. Unpaired *t*‐test was performed to test the difference of either FSC‐A or SSC‐A (scattering plots) between CD4^+^ T and CD8^+^ T cells (500 cells each), or B and NK cells (500 cells each), or T_N_ and non‐T_N_ cells (500 cells each). ***p < .001, ****p < .0001 (a,b, scatter plots). Meanwhile, Cohen's *d* (C*d*) was calculated and shown correspondingly (a,b, scatter plots). The average fold change for each lymphocyte subpopulation among SSC^high^ and SSC^low^ clusters is indicated as colored numbers (n = 4) (a,b). c–g) Unstained PBMC samples (n = 6) were equally sorted into SSC^high^ and SSC^low^ populations before lymphocyte subtypes determination as that in a and b. The average fold change for each lymphocyte subpopulation among SSC^high^ and SSC^low^ clusters is indicated as colored numbers (n = 6) (d–g). h) Expression profiles of selected phenotypic markers in the same T‐cell subsets but derived from SSC^high^ or SSC^low^ cell cluster (n = 2). i,j) Expression level of CCR7 (i) and CD45RA (j) as represented by relative fluorescence intensity (RFI) in CD4^+^ T or CD8^+^ T naïve/memory subsets from sorted SSC^high^ and SSC^low^ cells (n = 6). *p < 0.05, **p < 0.01, ***p < 0.001, ****p < 0.0001, paired two‐tailed *t*‐test (d‐g,i,j).

We hypothesized that the differences in cell granularity between functional lymphocyte populations, previously observed in high‐resolution methods such as electron microscopy^[^ ^18^ ^]^ or 3D‐refractive index tomography,^[^ ^19^ ^]^ could be detected in the high‐throughput SSC measurements collected by flow cytometry. To investigate this, we first pre‐stained PBMCs with lineage‐specific markers to identify CD4^+^ T, CD8^+^ T, NK, and B cells (Figure S1a, Supporting Information), and additional naïve/memory markers CD45RA/CCR7/CD45RO to identify T‐cell differentiation subsets (Figure S1b, Supporting Information). We found that CD8^+^ T and NK cells were enriched in lymphocytes at high SSC intensity, with correspondingly fewer CD4^+^ T cells (Cohen's *d* > 0.8, **Figure** **2**a; Figure S2a–d, Supporting Information). Notably, there was an even more apparent SSC difference between naïve and differentiated T‐cell subsets. Specifically, with increasing SSC intensity, there were more T_CM_, T_EM,_ and T_EMRA_ but fewer T_N_ cells (Cohen's *d* > 0.8, Figure 2b; Figure S2e‐j, Supporting Information). On the contrary, FSC was much less discriminative between lymphocyte subpopulations (Cohen's *d* < 0.5, Figure S3, Supporting Information). These findings demonstrate that functional subpopulations in lymphocytes can be distinguished by granularity using high throughput flow cytometry analysis.

The intrinsic differences in SSC suggested a way to enrich specific functional populations of cells. We confirmed that the SSC was minimally affected by sample staining procedures and voltage changes during data acquisition (Figure S4, Supporting Information), suggesting that this cell attribute can be reproducibly measured across different instrument platforms. In order to characterize the cells with high and low side scatter respectively, we defined the SSC^high^ population as cells that have SSC measurement above the median SSC value of the lymphocyte gate, and SSC^low^ populations as cells of the remained counterpart of lymphocyte (Figure 2c). We sorted unstained cells into SSC^high^ and SSC^low^ populations and then performed cell surface marker staining to identify their phenotype and differentiation state. We found that the SSC^low^ group is significantly enriched for CD4^+^ T and T_N_ cells, while the SSC^high^ cluster is significantly enriched for CD8^+^ T, NK, and other more‐differentiated T cells including T_CM_, T_EM,_ and T_EMRA_ (Figure 2d–g), confirming our previous results. Therefore, our results show that cell side scatter, as a reflection of the global cellular granularity, could be a robust biophysical property used for label‐free enrichment of lymphocyte subpopulations.

Compared to SSC^high^ T cells, the T cells from SSC^low^ population displayed a higher expression of costimulatory receptors such as CD27 and CD28 but a lower expression of differentiation marker CD127, memory marker CD45RO and activation/senescence factors CD95/CD57^[^ ^1^, ^3^ ^]^ (Figure S5a, Supporting Information), consistent with a larger number of T_N_ cells in this cell cluster but more terminally‐differentiated T cells in SSC^high^ cluster. Unexpectedly, both T_N_ and T_CM_ from the SSC^low^ group had reduced CD127, CD95, and CD57 when compared to those of the same lineages from the SSC^high^ group (Figure 2h). Also, T_CM_ SSC^low^ cells had an increased proportion of cells expressing CD27 (Figure 2h), and CCR7/CD45RA expression per cell (Figure 2i,j), suggesting its early T_CM_‐like phenotype. Functionally, in response to a short pulse of protein kinase C (PKC) stimulus, a larger proportion of SSC^low^ T cells produced IL‐2 but a lower proportion produced immediate effector cytokines that include IFN‐γ (Figure S5b, Supporting Information), resembling cytokine expression dynamics of early‐differentiated T cells.^[^ ^29^, ^35^ ^]^ Taken together, we found that PBMCs can be segregated into two functional groups according to their cellular granularity. Cells with high granularity (SSC^high^) are biased towards terminal differentiation and represent the immune arm that can exert immediate effector functions including cell cytotoxicity and secretion of pro‐inflammatory cytokines. On the other hand, cells with low granularity (SSC^low^) consists of a less differentiated population that is crucial for immunosurveillance but nevertheless highly adaptive to undergo further maturation upon antigen encounter.

### 2.2 Cellular Products Derived from SSC^high^ and SSC^low^ Lymphocytes Have Distinct Proliferative and Functional Capabilities

**Figure 3:**
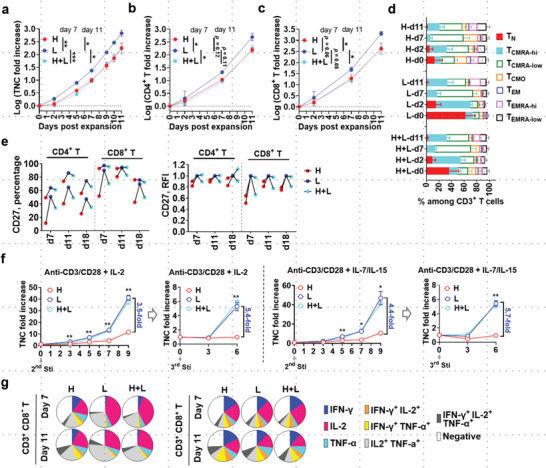
SSC‐sorted T cells have distinct proliferative and functional capabilities. a–c) Expansion efficiency of lymphocytes derived from SSC^high^ (“H”), SSC^low^ (“L”), or reconstituted SSC^high+low^ (“H+L”) cells at a ratio of 1:1. Data are mean ± S.E.M (n = 3), two‐tailed and paired *t*‐test for the data sets of day 7 and 11 respectively. *p < 0.05, **p < 0.01, ***p < 0.001. Exact *p*‐values for moderate but non‐significant trends were also indicated. d) Compositional change of naive/memory subsets in CD4^+^ T or CD8^+^ T cells over time. Data are mean ± S.E.M (n = 3). e) CD27 expression percentile and abundance as expressed by relative fluorescence intensity (RFI). f) Responsiveness of effector cells derived from SSC^high^, SSC^low^, or SSC^hi+low^ group at day 11 to multi‐stimulation by CD3/CD28 engagement in the condition of IL‐2 or IL‐7 plus IL‐15 supplemented. Duplicates for each sample were performed. Data are mean ± S.E.M. *p < 0.05, **p < 0.01, unpaired two‐tailed *t*‐test. g) Multifunctional cytokine expression of total CD8^−^ T or CD8^+^ T cells at day 7 and day 11 post‐expansion (n = 3).

Sustained remission of malignant tumors is among the central goals of redirected T cells‐based immunotherapy. Recent data suggest that T cell products derived from less‐differentiated T cells were shown to have better clinical outcomes in adoptive immunotherapy.^[^ ^23^, ^24^, ^26^, ^27^ ^]^ The functional dichotomy of immune cells segregated by cell granularity prompted us to investigate if these populations can be further expanded to yield cellular products of different potencies. We first investigated how the cells sorted by SSC intensities may differ in proliferation capability. In response to stimulation through CD3/CD28, SSC^low^ cells had three‐ to fourfold higher expansion of total nucleated cells (TNC), CD4^+^ T cells, and CD8^+^ T cells compared to the SSC^high^ group (**Figure** **3**a–c). Unexpectedly, the expansion of SSC^low^ cells was also significantly higher (3–4‐fold for CD4^+^ or CD8^+^ T, and 3‐fold for TNC) than SSC^high+low^ cells, suggesting that more cells can be derived from the half of the lymphocyte population with lower SSC than the entire lymphocyte population. A possible explanation for this could be precocious differentiation of T_N_ into less proliferative cell types driven by effector/memory T cells from the SSC^high^ compartment,^[^ ^36^ ^]^ and inhibitory effects on T cell proliferation from NK cells.^[^ ^37^ ^]^ Our results show the deleterious effect of the SSC^high^ cells on the overall expansion potential, and their removal has a net benefit not only to improve the cell quality but also the cell quantity during immunotherapy cell manufacturing.

Phenotypically, an in‐depth gating strategy was used to classify T‐cell subsets into T_N_, T_N_‐like CD45RA^high^ T_CM_ (T_CMRA‐hi_),^[^ ^38^ ^]^ CD45RA^low^ T_CM_ (T_CMRA‐low_), CD45RA^negative^ T_CM_ (T_CMO_), and T_EM_ with CD45RA expression as CD45RA^high^ T_EM_ (T_EMRA‐hi_), CD45RA^low^ T_EM_ (T_EMRA‐low_) and CD45RA^negative^ T_EM_ (T_EM_) (Figure S6a, Supporting Information). T cells expanded from SSC^low^ cells, as opposed to that expanded from SSC^high^ cells, had a higher proportion of T_CMRA‐hi_ but a lower proportion of T_CMRA‐low_ and other more‐differentiated cell subsets (e.g., T_CMO_ and T_EMRA‐low_) (Figure 3d; Figure S6b–e, Supporting Information). Besides, there was a continuously higher expression of persistence molecule CD27 in SSC^low^‐derived cells (Figure 3e), consistent with their greater proliferation capacity in response to multiple stimulations by anti‐CD3/CD28 microbeads (Figure 3f).

Progressive T‐cell differentiation has been linked to increased IFN‐γ production but weakened IL‐2 response,^[^ ^29^, ^39^ ^]^ as well as loss of TNF‐α secretion.^[^ ^35^ ^]^ On the other hand, the acquisition of full effector function by CD8^+^ T cells in vitro was shown to impair their antitumor efficacy in vivo where a persistent tumor‐suppressing capability is required for better survival.^[^ ^29^, ^39^ ^]^ The SSC^low^‐derived CD4^+^ T cells, and to a lesser extent CD8^+^ T cells, responded faster to short periods of PKC activation by producing more early‐effector cytokines (e.g., IL‐2^+^ and IL‐2^+^ TNF‐α^+^) and less late‐effector cytokines (e.g., IFN‐γ^+^, IFN‐γ^+^ IL‐2^+^, and IFN‐γ^+^ TNF‐α^+^) (Figure 3g; Figure S7, Supporting Information), suggesting that these SSC^low^‐derived effector cells are endowed with naïve‐like cytokine‐producing profiles.^[^ ^29^, ^35^, ^39^ ^]^ Therefore, cell descendants derived from the SSC^low^ cluster are characteristic of younger phenotypes and functionality that are beneficial for adoptive cell immunotherapy.

Of note, the cytokine secretion profile of T cells from donor 1 (“D1”) had an apparent difference compared to that of “D2” and “D3” (Figure S7b, Supporting Information), which could have resulted from their different composition at the beginning of cell expansion (Figure S6b‐d, Supporting Information). Specifically, the T‐cell source from “D1” had more T_N_ and less of those late‐differentiated counterparts, and this donor‐specific difference could lead to different cell functionalities after expansion.^[^ ^26^, ^29^ ^]^

### 2.3 Increased T_CMRA‐hi_ Derived from the SSC^low^ Cluster Resembles T_SCM_‐like T_CM_ Precursors

**Figure 4:**
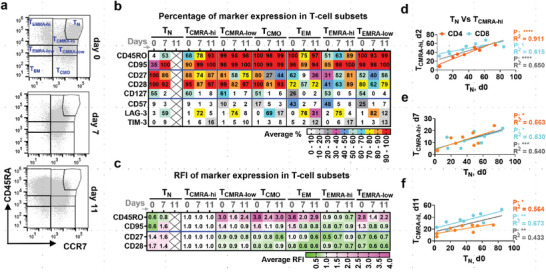
Increased T_CMRA‐hi_ derived from the SSC^low^ cell cluster resembles T_SCM_‐like central memory precursors. a) Gating strategy to identify T‐cell subset. b,c) Longitudinal assessment of phenotypic change of T_CMRA‐hi_ compared to other T‐cell subsets (n = 3). Average percentage expression (b) and abundance per cell (c) of selected phenotypic markers within T‐cell subsets were shown. The relative fluorescence intensity (RFI) was reported after normalization to that of T_CMRA‐hi_ for each time point. The cross label indicates omitted data due to low cell percentages/counts for marker expression determination or MFI calculation. d–f) Correlation analysis of starting T_N_ cell proportion with the percentage of generated T_CMRA‐hi_ after expansion. Data of SSC^high^, SSC^low^, and SSC^high+low^ from the same day were combined (n = 3). Each dot represents the percentage of produced T_CMRA‐hi_ versus that of initial cellular composition (day 0). The solid black line indicates linear regression of combined data. Two‐tailed F‐test, *p < 0.05, **p < 0.01, ***p < 0.001, ****p < 0.0001 (d–f).

The high expansion rate of SSC^low^ lymphocytes and the less differentiated phenotypes of cells being produced lead us to postulate the presence of prolific and potentially self‐renewing T‐cell subpopulations in this compartment. Upon further inspection, it was found that the T_CMRA‐hi_ cells shared the phenotypic traits (CCR7^+^ CD45RA^high^ CD45RO^+^ CD95^+^ CD28^+^ CD27^+^) as T_SCM_ (**Figure** **4**a‐c), regardless of CD4^+^ T or CD8^+^ T cells (Figure S8, Supporting Information). The expression of inhibitory/senescent factors (CD57 and LAG3) was comparable between CD8^+^ T_CMRA‐hi_ and CD8^+^ T_N_ but lower than other CD8^+^ T cell subsets (Figure S8c, Supporting Information). T_CMRA‐hi_ also differed from T_CMRA‐low_ and T_CMO_ by having a lower expression of CD45RO (memory marker) and a higher expression of CD27 (persistence marker). T_SCM_ cells have been recognized as multipotent progenitors derived from T_N_ that can both self‐renew and replenish more differentiated subsets of memory T cells,^[^ ^31^ ^]^ contributing to long‐term CAR‐T persistence in patients.^[^ ^25^ ^]^ Thus T_SCM_ –like T_CMRA‐hi_ cells in the SSC^low^ compartment could potentially serve as a renewable source of various effector T cells during expansion.

Interestingly, the abundance of T_CMRA‐hi_ cells in the SSC^low^ compartment was not high immediately post‐sorting. Instead, their abundance peaked in the short term (2 days) post‐expansion and continued in the long term (11 days) (Figure 3d). To investigate the origins of T_CMRA‐hi_ cells, we found that the abundance of T_CMRA‐hi_ throughout the expansion time course was strongly correlated with the initial proportion of T_N_ (Figure 4d‐f). This suggests that the T_SCM_‐like T_CMRA‐hi_ population could have derived from T_N_ cells shortly after expansion and is responsible for the overall proliferative capacity of the T cells. Taken together, pre‐enrichment of T_N_ cells in the SSC^low^ population enables efficient generation of T_CMRA‐hi_ cells that ensures a proliferative T‐cell population upon expansion.

### 2.4 CD4^+^ T Contributes to Optimal CD8^+^ T Expansion and T_CMRA‐hi_ Generation

Cell‐cell interaction is key to proper immune cell differentiation and proliferation. For example, help from CD4^+^ T cells is critical for the activation and persistence of CD8^+^ T cells,^[^ ^40^ ^]^ such as the ability to enhance tumor eradication by CD8^+^ T cells.^[^ ^41^ ^]^ As a corollary of the different expansion rates of CD4 and CD8 cells in vitro, a skewed CD4:CD8 ratio after expansion compared to pre‐manufacturing cells was observed (Figure S9a, Supporting Information). To investigate the effects of CD4:CD8 ratios in T cell proliferation and differentiation, we sorted pure CD4 and CD8 cells, combined them at defined ratios, and tracked their proliferation and differentiation during expansion (Figure S9b, Supporting Information).

**Figure 5:**
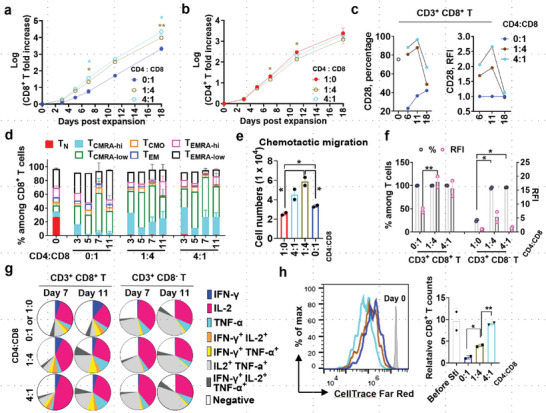
CD4^+^ T help is required for optimal CD8^+^ T expansion and central memory maintenance. a,b) Proliferation of CD8^+^ T and CD4^+^ T cells in the condition of co‐culture. Data from days 5, 7, 11, and 18 represent two independent experiments. c) Dynamic expression of CD28 as represented by percentile and RFI between cell groups. d) Compositional change of CD8^+^ naive/memory subsets over time. Data from days 0, 7, and 11 are mean ± S.E.M of two independent experiments. e) CCL21/CCL19‐driven migration capacity of T cells generated at day 11. f) Granzyme B expression of expanded T cells (day 7). RFI indicates the relative fluorescence intensity. Data are mean ± S.E.M of two independent experiments. g) Multifunctional cytokine expression of CD8^+^ T or CD4^+^ T cells at day 7 and day 11 post‐expansion. h) Resting CD4^+^ T cells were sorted and spiked into CellTrace dye‐labeled T cell products (day 13) at the indicated ratio and co‐stimulated (cells to beads = 1:1) for 4 days. Duplicates for each group were performed. Cells were counted on day 4 before flow cytometry analysis. Data are mean ± S.E.M. *p < 0.05, **p < 0.01, unpaired two‐tailed *t‐*test (a,b,e,f,h).

We found that CD8^+^ T cell proliferation was significantly improved in the presence of CD4^+^ T cells by an average of 6‐fold increase at day 7 and 8.5‐fold increase at 18 (**Figure** **5**a). On the other hand, CD4^+^ T cell proliferation was modestly inhibited (< 2‐fold) by CD8^+^ T cells when present in a mixture (Figure 5b). The results suggest that a high ratio of CD4:CD8 cells could benefit the overall cell expansion rate, as high amounts of CD4^+^ T cells improve the proliferation of CD8^+^ T cells and low amounts of CD8^+^ T cells provide less inhibition to CD4^+^ T cells growth. The results are consistent with our observation that the SSC^low^ population, with a high CD4:CD8 ratio, has a considerable growth advantage compared to the SSC^high^ and unsorted populations.

The availability of CD4^+^ T cell help also improved the quality of CD8^+^ T cells. Among a panel of cell function‐associated markers (Figure 5c; Figure S10, Supporting Information), CD28, a co‐stimulatory receptor that is required for CD8^+^ T function, was significantly upregulated in CD8^+^ T cells in the presence of CD4^+^ T cells in a dose‐dependent manner (Figure 5c). Meanwhile, longitudinal assessment of T‐cell differentiation subsets showed that CD8^+^ T_CMRA‐hi_ cells, which have been shown to be a T_SCM_‐like progenitor population, were significantly increased in the presence of CD4^+^ T cells (Figure 5d). The presence of CD4^+^ T cells is observed to be important but not dose‐dependent for yielding optimal CD8^+^ T cell composition during expansion. Further evidence of improved functionality of CD8^+^ T cells with CD4^+^ T cells help is the improved chemotactic migration of CD8^+^ T cells (Figure 5e), improved granzyme B expression (Figure 5f), and enhanced IL‐2 /TNF‐α but reduced IFN‐γ production (Figure 5g) indicating their less‐differentiated functional state. Moreover, resting CD4^+^ T cells were able to rescue the expansion potency of extensively differentiated CD8^+^ T cells (Figure 5h). Therefore, despite the absence of cognate antigens, CD4^+^ T cell help is critical for shaping CD8^+^ T cells’ functionality.

Taken together, there is substantial advantage to being able to maintain or even increase CD4:CD8 ratios in cell manufacturing procedures to improve the yield and quality of cell therapy products. As a natural consequence of different in vitro proliferation rates of CD4^+^ T and CD8^+^ T cells, the CD4:CD8 ratio could drop to very low values during prolonged expansion. Selection of the SSC^low^ population for cell manufacturing could represent an excellent strategy to achieve a favorable CD4:CD8 ratio post‐manufacturing. Alongside the ability to yield T cells of younger phenotypes, this approach could provide a single‐step solution to simultaneously enhance immunotherapy cell manufacturing on multiple fronts.

### 2.5 SSC^low^‐Derived CAR‐T Enhances Recursive Antileukemic Activity In Vitro

**Figure 6:**
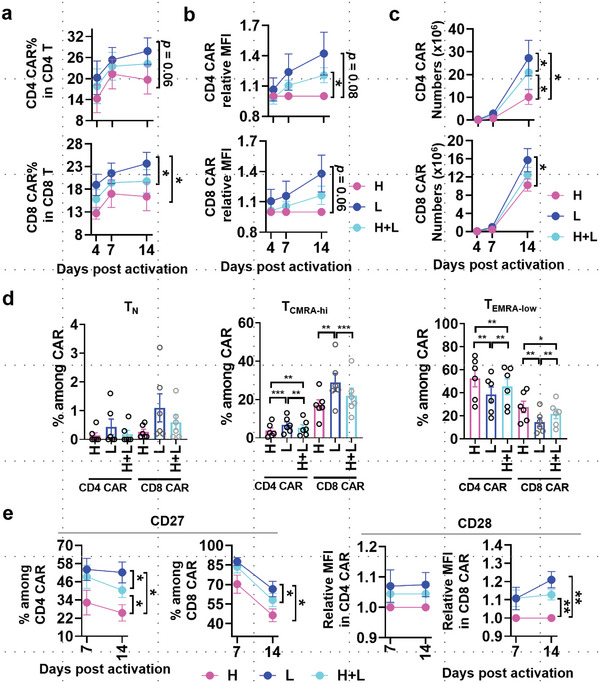
SSC^low^‐derived CAR‐T possesses favorable phenotypic profiles. a) Transduction efficiency of CAR‐expressing lentiviral vectors. b) CAR transgenic stability as represented by GFP abundance per cell among CAR‐expressing CD4^+^ T or CD8^+^ T cells derived from lymphocytes initially with different SSC intensity. c) Absolute CD4^+^ CAR‐T and CD8^+^ CAR‐T numbers increased over time. d) Percentage of T_N_, T_CMRA‐hi,_ and T_EMRA‐low_ among CD4^+^ CAR‐T or CD8^+^ CAR‐T cells at day 14 after expansion. e) Dynamic expression of CD27 and CD28 in generated CAR‐T cells. The relative MFI was shown after normalization to marker expression of the SSC^high^ group. Data are mean ± S.E.M (n = 6). *p < 0.05, **p < 0.01, ***p < 0.001, paired two‐tailed *t‐*test (a–e).

Next, we tested whether the desirable traits of SSC^low^ cells can be translated into practical benefits in the production of CAR‐T cells. We found that T cells derived from the SSC^low^ cluster had a higher lentiviral transduction efficiency (**Figure** **6**a; Figure S11, Supporting Information) and enhanced transgenic stability over the two weeks in vitro expansion course (Figure 6a,b), consistent with previous reports showing that T_N_ cells have higher expression of transduced genes encoding T‐cell receptors.^[^ ^26^ ^]^ In addition, CAR‐T cells derived from SSC^low^ cells (CAR.SSC^low^) contained a higher proportion of less‐differentiated CAR‐T subsets (e.g., T_N_ and T_CMRA‐hi_) and a lower proportion of late‐differentiated ones (e.g., T_EMRA‐low_) (Figure 6c,d; Figure S12, Supporting Information). Moreover, CAR.SSC^low^ possessed a higher expression of co‐stimulatory receptors like CD27 and CD28 (Figure 6e). Thus, SSC^low^ cells‐derived CAR‐T cells possess phenotypic properties that are desirable for immunotherapy.

**Figure 7:**
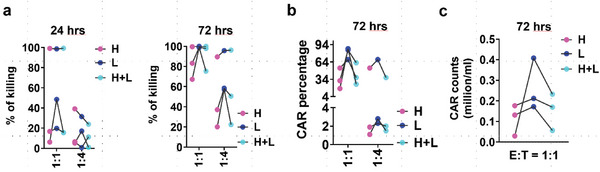
SSC^low^‐derived CAR‐T cells possess superior target‐killing potency in vitro. a) CAR‐T and NALM6.Luc cells were co‐cultured in 24‐well flat‐bottom culture plates at low E:T ratio (n = 3). Killing efficiency was calculated according to the reduction of luciferase intensity in the presence of CAR‐T compared to NALM6.Luc cells cultured alone. b) CAR‐T cell percentage over total cells in the co‐culture system at day 3. c) CAR‐T cell counts at day 3.

Functionally, CAR‐T cells derived from all three side scatter populations (CAR.SSC^high^, CAR.SSC^low^, and CAR.SSC^high+low^) were effective in target cell killing at high effector‐to‐target ratio (Figure S13a, Supporting Information). This is in line with clinical findings on the efficacy of CAR‐T therapy in controlling minimal residual disease in leukemia patients. We additionally observed that CAR.SSC^low^ cells were able to proliferate robustly after target clearance (Figure S13b, Supporting Information), which could be attributed to long‐lived memory cells. Immunotherapy becomes more challenging when T cells in vivo have to respond against a large number of tumor cells. As CAR‐T cells are prone to functional impairment, the acquisition of recursive killing potency of effector T cells becomes important.^[^ ^3^ ^]^ Thus, we adopted a killing assay at high tumor cell loads (low effector‐to‐target ratio).^[^ ^42^, ^43^ ^]^ As can be seen, CAR.SSC^low^ exhibited stronger growth inhibition of NALM6.Luc cells (**Figure** **7**a; Figure S14, Supporting Information), accompanied by the better proliferation of effector cells (Figure 7b,c). The differences in absolute CAR counts in Figure 7c are due to donor‐specific differences in cell proliferation rate, but the overall trend of highest proliferation in the CAR.SSC^low^ population remains consistent. These results imply the potential utility of using CAR.SSC^low^ for enhanced effector engraftment and persistence, facilitating control of tumor progression.

### 2.6 SSC^low^‐Derived CAR‐T Shows Enhanced Antileukemic Activity in Vivo

**Figure 8:**
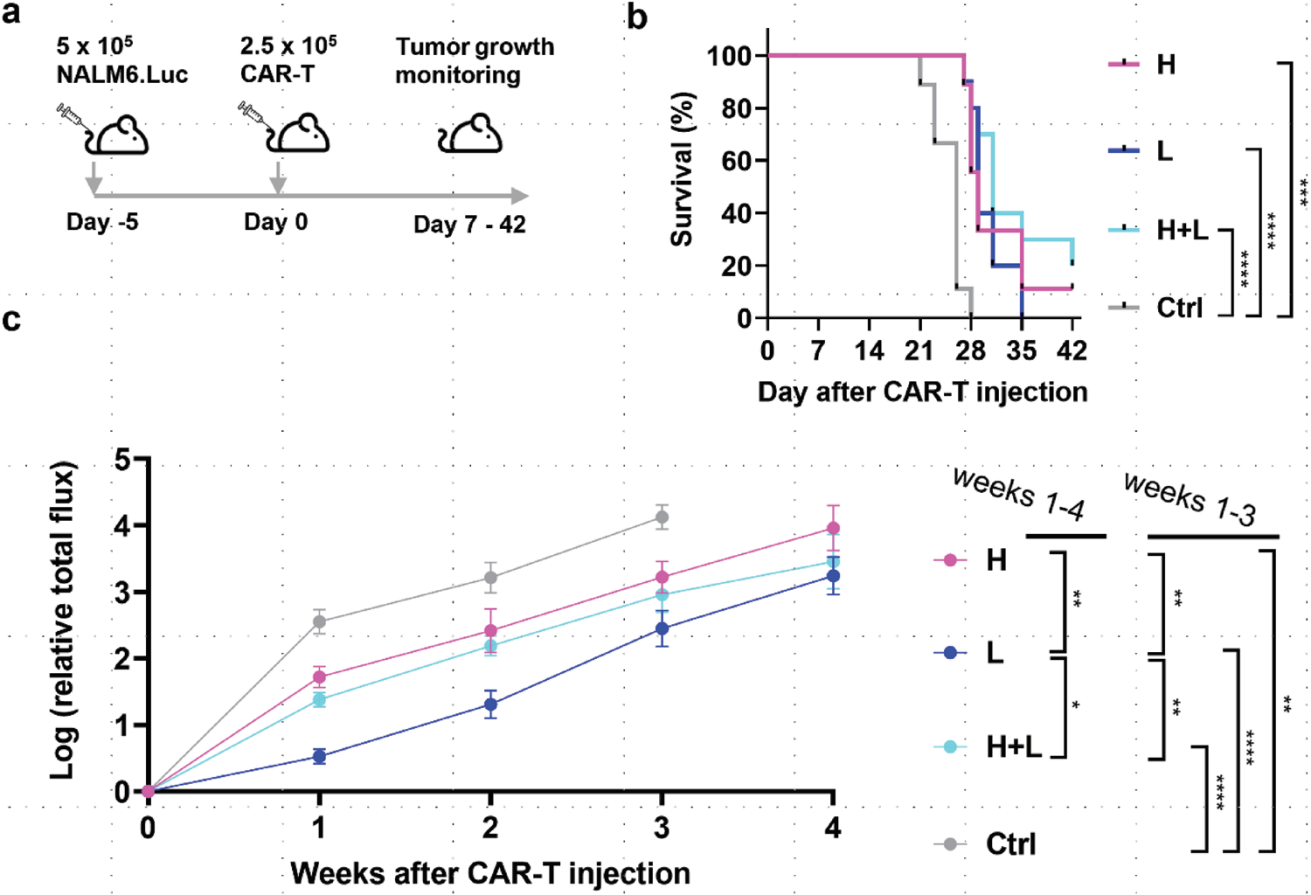
SSC^low^‐derived CAR‐T cells enhance target‐killing activity in vivo. a) NSG mice were intravenously injected with 5 × 10^5^ NALM6.Luc leukemic cells 5 days before treated with 2.5 × 10^5^ CAR‐T cells or control T cells without CAR expression (n  =  10 mice each for CAR.SSC^low^ and CAR.SSC^high+low^ group, and 9 each for CAR.SSC^high^ and control group). b) Kaplan–Meier survival analysis. *P*‐values were determined using a log‐rank test. c) Quantification of tumor burden from week 0 to week 4 by measuring total photon flux per second. Shown were normalized total flux to the value of week 0 for each mouse before CAR‐T infusion. Data are mean ± S.E.M, and two‐way ANOVA test was performed between two groups. Sidak's multiple comparisons test was performed following ANOVA analysis (Figure S16, Supporting Information). *p < 0.05, **p < 0.01, ***p < 0.001, ****p < 0.0001.

Finally, we tested whether the CAR‐T cells derived from cell sources of distinct SSC intensity would show differences in their antileukemic effects in vivo. NSG mice were injected with 5 × 10^5^ CD19^+^ luciferase‐expressing NALM6 cells (NALM6.Luc). After 5 days, tumor‐engrafted mice were injected with 2.5 × 10^5^ purified CAR‐T cells derived from different cell populations (10 mice each for CAR.SSC^low^ and CAR.SSC^high+low^ group, and 9 each for CAR.SSC^high^ and control group). This condition simulates the low effector‐to‐target ratio in clinics.^[^ ^44^ ^]^ Mice tumor loads were measured weekly over 6 weeks following CAR‐T injection to assess tumor progression (**Figure** **8**a).

Our results showed that there was a significant difference between the CAR‐T injected vs non‐CAR‐T injected mice (median survival 26 days vs 29 days, *p* < 0.0001), indicating that CAR‐T cells were effective in controlling tumor growth (Figure 8b). On the other hand, there was no statistically significant difference in the survival of mice injected with different CAR‐T populations. While these results suggest that long‐term benefits have not been achieved in this single‐dose treatment modality, tracking of the tumor growth over time revealed some surprising results. We considered the flux from weeks 1–4 as beyond that very few mice were alive (1 for the CAR.SSC^high^ group and 3 for CAR.SSC^high+low^ at week 5). The total photon flux reflecting the tumor cells was consistently and statistically significantly lower in mice injected with CAR.SSC^low^ cells compared to mice injected with CAR.SSC^high^ or CAR.SSC^high+low^ cells (Figure 8c; Figure S15, Supporting Information). This observation was consistent with *in‐vitro* results showing superior target killing of CAR.SSC^low^ cells (Figure 7). However, the tumor control advantage of CAR.SSC^low^ cells diminished in weeks 3 and 4 (Figure S15, Supporting Information). NSG mice lack essential human cytokines and other supportive elements (e.g., stromal cells) that can promote CAR‐T cell trafficking and proliferation.^[^ ^44^ ^]^ Insufficient expansion of CAR‐T cells in the xenograft mouse model could be one potential reason for the lack of long‐term benefit despite clear short‐term improvements. Given that CAR.SSC^low^ cells demonstrate superior initial tumor‐suppressing potential in vivo, multiple infusions of these populations may maximize their ability to control tumor growth over time.^[^ ^45^, ^46^ ^]^

## 3 Discussion

The form of a cell is closely related to its function. As early as the late 19th century, the field of diagnostic cytology which uses aberrant shapes of cells found in tissue samples as a marker for disease has emerged.^[^ ^47^ ^]^ As cellular heterogeneity is inevitable in all cell‐based analyses and therapies, a complex readout of the ensemble of cell states may be achieved through morphological analysis.

Studies have shown that different properties and structures of a cell affect the angular distribution of scattered light.^[^ ^48^ ^]^ Small‐angled forward scattered light (*θ* = 0–2°) is primarily dependent on the cell size and refractive index, while larger‐angle forward scattered light (*θ* = 5–30°) is strongly dependent on the nucleus of the cell. Side‐scattering at large angles (*θ* = 50–130°) is highly dependent on the small organelles in the cell such as mitochondria, peroxisomes, lysosomes, and granules. Finally, in the backscatter region (*θ* = 160–180°), the cell membrane is responsible for scattered light. The SSC measurement from flow cytometry is collected at *θ* = 90° and therefore is expected to provide rich information regarding cellular granularity, particularly due to organelles such as granules and mitochondria.

Our work demonstrates a strong association of immune cell functional subtypes with cellular granularity measured by SSC in flow cytometry. CD8^+^ T, NK, and late‐differentiated T cells have high side scatter, while CD4^+^ T, naïve, and early memory T cells are significantly enriched in the low side scatter population. Unlike a cell‐surface marker approach that is capable of selecting very specific molecular subtypes of cells, the biophysical property approach selects cells based on a convergence of functions. Cells in the SSC^high^ compartment are predominantly involved in immediate effector functions, while those in the SSC^low^ compartment play the roles of immune surveillance.

Recent studies have found that the composition of infused cell products in adoptive cell immunotherapy is a major determinant of leukemia treatment outcomes.^[^ ^23^, ^24^ ^]^ However, at the same time, preparation of a pure cell population (e.g., CD8^+^ T cells)^[^ ^49^, ^50^ ^]^ might not be the best way in therapeutic T cell manufacturing due to the lack of the requisite interactions (e.g., CD4^+^ T help) between different cell types.^[^ ^40^, ^41^ ^]^ Our biophysical sorting method to isolate cells that collectively promote specific immune functions represents a novel strategy for improving the therapeutic efficacy of immunotherapy. While selecting cells that have superior attributes for immunotherapy, maintenance of cellular diversity promotes cell proliferation while preventing precocious differentiation in the final cell therapy product.

In a proof of concept, we demonstrated that CAR‐T cells derived from the SSC^low^ group (CAR.SSC^low^) have significantly elevated CAR‐T_CMRA‐hi_ subpopulation (CCR7^+^ CD45RA^high^ CD45RO^+^ CD95^+^ CD28^+^ CD27^+^), which resembles T_SCM_‐like cells reported to be a critical subpopulation in persistent immune memory.^[^ ^38^ ^]^ In addition, CAR‐T produced from the SSC^low^ cluster was able to maintain not only a relatively high CD4/CD8 ratio but also improved antileukemic activity post extensive expansion in vitro. The use of a balanced ratio of CD4/CD8 CAR‐T products has shown preliminary success in antileukemic studies and has translated into an FDA‐approved CAR‐T cell product (lisocabtagene maraleucel or Breyanzi).^[^ ^49^, ^50^, ^51^, ^52^ ^]^ As many patients with cancers or chronic diseases tend to manifest a decreased ratio of CD4/CD8 T cells,^[^ ^49^, ^52^, ^53^, ^54^ ^]^ it is expected that cell therapy products manufactured from the SSC^low^ cluster may further potentiate therapeutic efficacy.

We showed that CAR.SSC^low^ cells have outstanding antileukemic activity in vitro. The advantages of CAR.SSC^low^ cells also translated to enhanced early control of tumor growth in NSG mice, as evident by the consistently lower bioluminescence flux in mice injected with CAR.SSC^low^ cells compared to other conditions. However, there were no statistically significant benefits in the survival of mice injected with CAR.SSC^low^ cells despite the promising early response. Several factors could potentially explain these contradictory results. First, the long‐term proliferation of CAR‐T cells in NSG mice could be limited due to a lack of essential cytokines and chemokines as well as other critical elements supporting cell trafficking and maintenance.^[^ ^44^ ^]^ Second, the in vivo effector‐to‐target ratio in these experiments was expected to be very low (5 × 10^5^ tumor cells grown for 5 days before injection of 2.5 × 10^5^ CAR‐T cells), this condition could promote CAR‐T cell exhaustion in later times. Finally, further differentiation of CAR.SSC^low^ cells could occur over extended periods in vivo due to extensive antigen encounters. Potential solutions to harness the enhanced initial antileukemic activity of CAR.SSC^low^ cells for durable response could include supplementation with proliferation‐inducing/homeostatic cytokines,^[^ ^55^, ^56^ ^]^ higher or multiple sequential doses of CAR‐T injection,^[^ ^45^, ^46^ ^]^ and shortening CAR‐T cell expansion duration to reduce their differentiation during cell manufacturing.^[^ ^57^, ^58^ ^]^

While we have demonstrated a simple separation of T cells into populations above and below the median optical side scattering value already yields remarkable advantages for improving manufacturing products of cell‐based immunotherapy, there are potentially many opportunities to further enhance this process. First, this process can be easily integrated with other label‐free methods such as flow‐cytometry‐based auto‐fluorescence measurement of mitochondria flavoproteins^[^ ^59^ ^]^ to select T‐cells with optimal metabolic profiles for further expansion. It would also be very interesting to consider whether more complex gating approaches on cell biophysical attributes can yield better cell populations for specific applications. For example, GateID^[^ ^60^ ^]^ is a computational method that combines single‐cell transcriptomics with FACS index sorting to define an optimal gate for purifying the cell types of choice. One could potentially adapt this approach to search for a gate based on SSC and FSC to achieve high purity of specific T‐cell subtypes (defined by surface phenotypes). As a proof of concept, we combined FSC and SSC measurements to search for the optimal gate for recovering CD4^+^ T, CD8^+^ T, T_N_, T_CM_, T_EM, and_ T_EMRA_ cells (Figure S17, Supporting Information). Our results show that specific cell populations can be selected based on sorting native cell properties alone, although high cell purity may sometimes be accompanied by reduced cell yield. The simple strategy presented in this manuscript (based on the 50^th^ percentile of SSC measurement alone) represents a practical and easily‐to‐implement method for improving the quality of source cells for cell‐based immunotherapy.

We have demonstrated the efficacy of our approach on a mouse model, using cells sorted on a FACS sorter. There are several additional requirements for this method to be adopted for human trials, but we believe that they are within reach in the near future. First, cell manufacturing for human applications has to be performed in a good manufacturing practice (GMP) facility. In this respect, it is timely that GMP‐compliant high‐performance cell sorters have become increasingly common and had been used for manufacturing cell‐based immunotherapy products for small‐scale human studies.^[^ ^61^, ^62^ ^]^ Secondly, the number of cells required for human applications (≈10^9–10^ cells) is typically far greater than in mouse studies (≈10^6–7^ cells). This is achievable as our approach sorts 50% of the cells and includes a further expansion step that increases target cell numbers by >1000‐fold. Sorting 1 × 10^7^ cells at a speed of 10 000 cells s^−1^ at a 50% positive rate would require ≈30–60 min, which is acceptable considering the entire workflow of cell manufacturing (several weeks). Even higher throughput cell sorters in the pipeline, such as those based on Vortex Actuated Cell Sorting (VACS, cell flow rate ≈2 × 10^5^  s^−1^),^[^ ^63^ ^]^ would further simplify and provide the impetus for using this approach for improving cell manufacturing for immunotherapy.

In conclusion, we have demonstrated that a label‐free cell sorting strategy could be beneficial for cell therapy product manufacturing while circumventing the limitations of antibody‐based approaches. Beyond side‐scattering, there are many other cellular biophysical attributes (e.g., morphology, metabolic activity, electrical properties, etc.) that would be interesting to explore as means to segregate cells with specific properties. We believe that this is a fertile ground for many innovations. In addition, this study represents an important advance in the development of CAR‐T‐manufacturing protocols for improved therapeutic index. Our results strongly suggest that, the preservation of CAR‐T functional fitness with regard to its persistent activity results from not only a high proportion of T_N_ and early T_CM_ but also a relatively high ratio of CD4/CD8 T cells before CAR‐T engineering, which can be simply achieved via T‐cell sorting based on SSC.

## 4 Experimental Section

### Primary Cells and Cell Lines

Apheresis residual blood cones were collected from anonymous healthy adult donors from the Health Sciences Authority (HSA), Singapore, with approval from Institutional Review Board and informed consent from donors (NUS‐IRB no. H‐18‐038E). Peripheral blood mononuclear cells (PBMC) were enriched by Ficoll Paque Plus (GE Healthcare) and cryopreserved. To ensure batch‐to‐batch consistency, the enriched PBMC were cryopreserved at 5–10 million cells per vial in media containing 90% fetal bovine serum (FBS) (Gibco) and 10% DMSO (Sigma‐Aldrich). For each experiment, the cells were recovered overnight in RPMI1640 (Gibco, A1049101) supplemented with 10% FBS before downstream experiments. NALM6.Luc, a B cell precursor leukemia cell line stably expressing firefly luciferase, was maintained in RPMI 1640 containing 10% FBS and used within one week.

### Flow Cytometry Antibodies

Flow cytometry antibodies used for surface marker staining are listed as follows. Antibodies from BD Biosciences include: CD3‐BUV395 (Clone UCHT1; cat #563 546), CD4‐BV786 (Clone SK3; cat #563 877) and CD8‐PE‐Cy7 (Clone SK1; cat #335 787). Antibodies from Biolegend include: CD3‐FITC (Clone UCHT1; cat #300 406), CCR7‐PE (Clone G043H7; cat #353 204), CD45RA‐APC (Clone HI100; cat #304 112), CD45RO‐Pacific Blue (Clone UCHL1; cat #304 215),CD27‐Pacific Blue (Clone M‐T271; cat #356 413), CD28‐FITC (Clone CD28.2; cat #302 906), CD28‐PE (Clone CD28.2; cat #302 907), CD95‐FITC (Clone DX2; cat #305 605), CD127‐Brilliant Violet 785 (Clone A019D5; cat #351 329), TIM‐3‐Pacific Blue (Clone F38‐2E2; cat #345 041), CD57‐FITC (Clone HNK‐1; cat #359 603), LAG‐3‐Brilliant Violet 785 (Clone 11C3C65; cat #369 321) and human TruStain FcX reagent (Cat #422 302). Antibodies for intracellular staining include granzyme B‐PE (BD Biosciences, GB11; cat #561 142), IFN‐γ‐FITC (Miltenyi Biotec, cat #130‐090‐433), IL‐2‐PE (Miltenyi Biotec, cat #130‐090‐487) and TNF‐α‐APC (Miltenyi Biotec, cat #130‐091‐267).

### Characterization of T‐cell Differentiation State

In this study, two gating methods were used to identify T‐cell differentiation subsets. Resting T cells can be broadly classified as naïve (T_N_, CD45RA^high^ CCR7^high^ CD45RO^−)^, central memory (T_CM_, CD45RA^dim/‐^ CCR7^low^), effector memory (T_EM_, CD45RA^dim/‐^ CCR7^−^) and terminally differentiated effector (T_EMRA_, CD45RA^high^ CCR7^−^)^[^ ^20^, ^64^ ^]^ based on the expression level of canonical markers CCR7/CD45RA/CD45RO. For T cells undergoing activation and proliferation, an in‐depth gating strategy was adopted where the T_CM_ is further dissected into CD45RA^high^ T_CM_ (T_CMRA‐hi_),^[^ ^38^ ^]^ CD45RA^low^ T_CM_ (T_CMRA‐low_) and CD45RA^–^ T_CM_ (T_CMO_), while T_EM_ with CD45RA expression was classified into CD45RA^low^ T_EM_ (T_EMRA‐low_) and CD45RA^high^ T_EM_ (T_EMRA‐hi_).

### Characterization of T‐Cell Activation/Senescence State

To better understand T‐cell activation/senescence state either before or during expansion, the expression profile of a panel of selected cell surface markers was longitudinally assessed, including classical naïve/memory markers CCR7/CD45RA/CD45RO, proliferation and survival‐enhancing costimulatory receptors CD27/CD28, and immune activation/inhibition markers CD127/CD95/CD57/LAG‐3/TIM‐3. Specifically, the staining panels were designed as follows: panel for dynamic change of naïve/memory composition (CD3‐FITC/CD4‐BV786/CD8‐PE‐Cy7/CCR7‐PE/CD45RA‐APC/CD45RO‐V450), panel for co‐expression of co‐stimulatory markers in naïve/memory subsets (CD3‐BUV395/CD8‐PE.Cy7/CCR7‐PE/CD45RA‐APC/CD27‐Pacific Blue/CD28‐FITC), and two panels for immune activation/inhibition profiling in naïve/memory T‐cell subsets (CD3‐BUV395/CD8‐PE‐Cy7/CCR7‐PE/CD45RA‐APC/CD45RO‐Pacific Blue/CD95‐FITC/CD127‐BV785 and CD3‐BUV395/CD8‐PE‐Cy7/CCR7‐PE/CD45RA‐APC/TIM‐3‐Pacific Blue/CD57‐FITC/LAG‐3‐BV785). Pre‐blocking step with TruStain FcX (BioLegend) was included for all staining. For some experiments where dead cells may be generated such as the first 2–3 days post T cell activation and PMA‐based stimulation, LIVE/DEAD viability dye (Invitrogen, L34963) was used to exclude dead cells. Flow cytometry compensation matrices and data acquisition were completed in CytoFLEX analyzer (Beckman Coulter) and analyzed by FlowJo software version 10.

### Label‐Free Cell Sorting

Overnight‐recovered PBMCs were briefly washed and resuspended in cold phosphate buffer (PBS) before equally sorted into SSC^high^ and SSC^low^ populations on Moflo Astrios cell sorter (Beckman Coulter). The excitation wavelength for the generation of SSC (488/6‐nm filter) and FSC (488/6‐nm filter) was 488 nm. In this study, SSC was measured at an orthogonal angle from the light beam propagation axis while FSC was measured anti‐parallel to the incident beam. Where indicated, the sorted cells were further stained to characterize the composition of CD3^+^ CD4^+^ T, CD3^+^ CD8^+^ T, NK cells, B cells, and/or T‐cell subsets including T_N_, T_CM_, T_EM_, and T_EMRA_.

### Cell Enrichment by Automatic Gate Search

GateID,^[^ ^60^ ^]^ a computational algorithm for automatic gate search, was used to find the optimal gate for enriching cells of interest from a mixed cell population. Briefly, PBMC was recovered overnight and stained with indicated antibody panels for T‐cell characterization (CD3‐BUV395/CD4‐BV786/CD8‐PE‐Cy7/CCR7‐PE/CD45RA‐APC). The raw readouts corresponding to FSC‐A and SSC‐A parameters of each T‐cell subtype were exported and analyzed by the GateID package. The yield of the target cell type was set to 5% – 100% and gating vertices were constantly set to 4. The yield is the count of target cell type within the gate to that among the whole cell population, while the purity indicates the proportion of target cell type within the gate.

### Characterization of T‐cell Effector Factors‐Producing Capabilities

To determine the capabilities of T cells in cytokine or granzyme B expression, after removal of residual anti‐CD3/CD28 microbeads, the expanded T cells at day 11 were re‐stimulated by 50 ng mL^−1^ PMA and 1 µg mL^−1^ ionomycin (Sigma–Aldrich) for additional 2.5 h after removal of residual anti‐CD3/CD28 microbeads. Cells were collected and labeled with Live/Dead viability dye before being further blocked with TruStain FcX and stained with CD3‐BUV395 and CD8‐PE‐Cy7. After fixation and permeabilization using Cytofix/Cytoperm reagent (BD Biosciences), the sample was divided into two tubes for either cytokine detection with IFN‐γ‐FITC, IL‐2‐PE, and TNF‐α‐APC or granzyme B measurement with granzyme B‐PE.

### T cell Activation and Expansion In Vitro

Label‐free sorted SSC^low^ and SSC^high^ cells were either expanded alone or reconstituted at a ratio of 1:1 (equal to total lymphocytes within the initial cell sample) for co‐expansion. The starting cell numbers for each condition were 0.06–0.08 million (experiment dependent) per well in 96‐well plates. After 2–4 hours’ incubation at 37 °C, all cell groups were activated using anti‐CD3/CD28 Dynabeads (3:1 bead‐to‐cell ratio) (Gibco) with 5 ng mL^−1^ IL‐2 (Gibco, PHC0021) supplemented. In some experiments, the expanded T cells were re‐stimulated by anti‐CD3/CD28 microbeads at a 1:1 bead‐to‐cell ratio under 5 ng mL^−1^ IL‐2, or 5 ng mL^−1^ each of IL‐7 and IL‐15 (PeproTech). Half of the culture medium was replaced with fresh medium containing 5 ng mL^−1^ IL‐2 every two days during initial activation/expansion stages (day 3 to day 7) and daily during late expansion phases (day 7 to day 11 or more where indicated). Cells were counted by trypan blue exclusion and split into ≈0.1 million per well. Compositional changes of T‐cell differentiation subsets and functionality as well were determined at indicated time points post‐activation.

### CD4^+^ T‐CD8^+^ T Co‐Culture Assay

PBMC were pre‐blocked with TruStain FcX and stained with CD4‐BV786 and CD8‐PE‐Cy7 for ensuing separation of CD4^+^ cells and CD8^+^ cells on Moflo Astrios cell sorter. Notably, only cells with the highest CD8 staining were sorted as CD8^+^ T cells due to diminished CD8 expression on some NK cells. Also, anti‐CD3 was not used for cell sorting where the sorted cells would be subjected to CD3/CD28‐engaged stimulation. The sorted CD4^+^ and CD8^+^ cells were validated to achieve sufficient purity (> 97%) and reconstituted at indicated ratios to achieve 0.06 million per well in 96‐well plates before activation/expansion as described before. To assess the ability of CD4^+^ T cells to rescue the proliferation capability of extensively pre‐expanded CD8^+^ T cells, cell materials derived from the SSC^high^ group (day 13) which contained a large number of terminally differentiated CD8^+^ T cells were labeled with Celltrace Far Red (Invitrogen) following the manufacturer protocol. The labeled CD8^+^ T cells and sorted resting CD4^+^ T cells of the same donor were reconstituted at indicated ratios and re‐stimulated by anti‐CD3/CD28 microbeads at a 1:1 bead‐to‐cell ratio under 5 ng mL^−1^ IL‐2. After 4 days, total cell numbers were counted and CD8^+^ T cell proliferation was detected by flow cytometry.

### In Vitro Migration Assay

In vitro chemotactic migration was used to evaluate T cell lymphoid tissue‐homing capacity. Briefly, CCL19 and CCL21 (300 ng mL^−1^ each) (PeproTech) in 100 µL culture medium were added to the lower chamber of a 96‐well transwell plate (5 µm porosity) (Corning, CLS3388). Expanded T cells (0.1 million) as indicated were seeded into the upper chamber in an 80 µL culture medium. After 5 h, the number of cells migrating to the lower chamber was counted by trypan blue exclusion.

### CAR‐T Cells Generation

A third‐generation lentiviral vector construct encoding a second‐generation 4‐1BB‐CD3‐zeta anti‐CD19 CAR with GFP expression downstream of an IRES (CAR.19‐4‐1BBz‐IRES‐GFP) was used.^[^ ^65^ ^]^ The lentiviral vector was produced by transient transfection of Lenti‐X 293T Cell Line (Takara Bio Inc.) using Lipofectamine 3000 (Thermo Fisher), followed by concentration of the lentiviral supernatant by ultracentrifugation. Label‐free sorted cells were rested for 4–12 h before being activated by anti‐CD3/CD28 microbeads (2:1 bead‐to‐cell ratio) for additional 24 h in 24‐well plates in AIM‐V medium (2% human AB serum). Stimulated cells were then transduced with CAR‐encoding lentivirus at a multiplicity of infection (MOI) of 5.0. RetroNectin‐coated 24‐well plate and spinoculation at 1000 × *g* for 60 min at 25 °C were used to enhance transduction. The viruses were removed after 3 days post‐transduction and the cells were expanded for 9–10 more days in G‐Rex 24‐well plates in AIM‐V medium supplemented with 5 ng mL^−1^ IL‐2. Half of the culture medium was changed with fresh medium containing 5 ng mL^−1^ IL‐2 at an interval of 3–4 days. The expanded CAR‐T cells were harvested and cryopreserved for future experiments.

### CAR‐T Antileukemic Activity in Vitro

GFP^+^ CAR‐T cells were sorted from expanded T cells (day 13) and rested at 37 °C overnight. To determine the immediate cytolytic activity of effector cells, a total of 0.21 million CAR‐T cells and 0.07 million NALM6.Luc cells were co‐incubated at a ratio of 3:1 (representative of low tumor cell loads) in a 96‐well U‐bottom plate. Meanwhile, to determine the long‐term tumor‐inhibiting effect of effector cells, a total of 0.2 million or 0.05 million CAR‐T and 0.2 million NALM6.Luc was co‐cultured, equal to a ratio of 1:1 or 1:4 respectively (representative of high tumor cell loads), in a 24‐well flat‐bottom plate. Killing dynamics over time were measured by luminescence detection (Promega, E6110). The target‐killing efficiency for CAR‐T cells in the co‐culture conditions was represented as the percentage of reduced luminescence readout compared to that without CAR‐T cells added, wherein NALM6.Luc cells continued to proliferate. As indicated on day 3 post‐co‐culture, absolute cell numbers were manually counted by trypan blue exclusion, meanwhile, part of the cells were aspirated out and subjected to flow cytometry analysis for the determination of remaining CAR‐T cells in the co‐culture system.

### CAR‐T Antileukemic Activity In Vivo

All animal work was conducted in accordance with the A*STAR‐approved IACUC protocol (#221 738). NOD‐*scid IL2rγ^null^* (NSG) mice purchased from the Jackson Laboratory (stock #0 05557) were bred and kept under conditions free of specific pathogens with 12 hours of light‐dark cycle. To evaluate the antileukemic activity of CAR‐T cells, NSG mice (8–10 weeks old) were engrafted with 5 × 10^5^ luciferase‐expressing acute lymphoblastic leukemia cell line, NALM6 (NALM6.Luc), via tail vein. GFP^+^ CAR‐T cells, generated from lymphocyte populations of different SSC intensities, were enriched by FACS (Moflo Astrios cell sorter, Beckman Coulter). The respective CAR‐T cells were rested at 37 °C overnight before infusing 2.5 × 10^5^ cells per mouse via the tail vein on day 5 after NALM6.Luc engraftment. Control mice were infused with GFP^−^ T cells. Tumor progression was monitored using the IVIS Spectrum in vivo imaging system (PerkinElmer) weekly. Briefly, 150 mg kg^−1^ luciferin was injected intraperitoneally into each mouse, the signal was quantified after 10 minutes and analyzed using the Living Image Software (PerkinElmer). The total photon flux measured was adjusted for the background signal and normalized to that of week 0 pre‐treatment. For survival analysis, in addition to actual death, weight loss of more than 20% pre‐treatment body weight was regarded as a death event.

### Statistical Analysis

All values with error bars are reported as mean ± SEM. Statistical analysis was performed using GraphPad Prism software (v8.0.2). Unpaired two‐tailed *t*‐tests were used to evaluate the statistical significance between two groups. Where indicated, paired two‐tailed *t*‐test was used instead. Two‐tailed F‐test was performed for linear regression. Log‐rank test was performed between two groups for Kaplan–Meier survival curve. Two‐way ANOVA tests were performed between two groups with Sidak's multiple comparisons post‐hoc test. Effect sizes were measured by Cohen's *d* given by the formula C*d* = (*M*_2_ – *M*_1_) ⁄ *SD*_pooled_, where *M* denotes the mean of each distribution and *SD*_pooled_ denotes the pooled standard deviation. A Cohen's *d* of 0.2, 0.5, and 0.8 was commonly used to represent small, medium, and large effect sizes.

## Conflict of Interest

The authors declare no conflict of interest.

## Author Contributions

T.W. and J.H.L.T. contributed equally to this work. T.W. and L.F.C. conceived and designed the study. T.W. performed the experiments and data analysis with assistance from W.X.S. (M.E.B.’s group) in the generation of CAR‐T cells, and input from J.H.L.T., S.Y.T., and M.G. (Q.C.’s group) in the mouse work. T.W. and L.F.C. wrote the manuscript draft. W.X.S., Y.H.L., M.E.B., J.H.L.T., and Q.C. reviewed the manuscript and provided feedback. All authors commented on the manuscript and approved the submission.

## Supporting information

Supporting Information

## Data Availability

The data that support the findings of this study are available in the supplementary material of this article.
