# Supplementary material for: Cell Granularity Reflects Immune Cell Function and Enables Selection of Lymphocytes with Superior Attributes for Immunotherapy
Source: Adv Sci (Weinh). 2023 Aug 6;10(28):2302175. doi: 10.1002/advs.202302175 (PMC10558660; doi:10.1002/advs.202302175)
Supplement: Supplementary file 1 — Supporting Information [file ADVS-10-2302175-s001.pdf]

ADVANCED SCIENCE
Open Access

Supporting Information

for *Adv. Sci.*, DOI 10.1002/advs.202302175

Cell Granularity Reflects Immune Cell Function and Enables Selection of Lymphocytes with Superior Attributes for Immunotherapy

Tongjin Wu, Joel Heng Loong Tan, Wei-Xiang Sin, Yen Hoon Luah, Sue Yee Tan, Myra Goh, Michael E. Birnbaum, Qingfeng Chen and Lih Feng Cheow**

Supporting Information

Cell granularity reflects immune cell function and enables selection of lymphocytes with superior attributes for immunotherapy

Tongjin Wu, Joel Heng Loong Tan, Wei-Xiang Sin, Yen Hoon Luah, Sue Yee Tan, Myra Goh, Michael E. Birnbaum, Qingfeng Chen, Lih Feng Cheow**

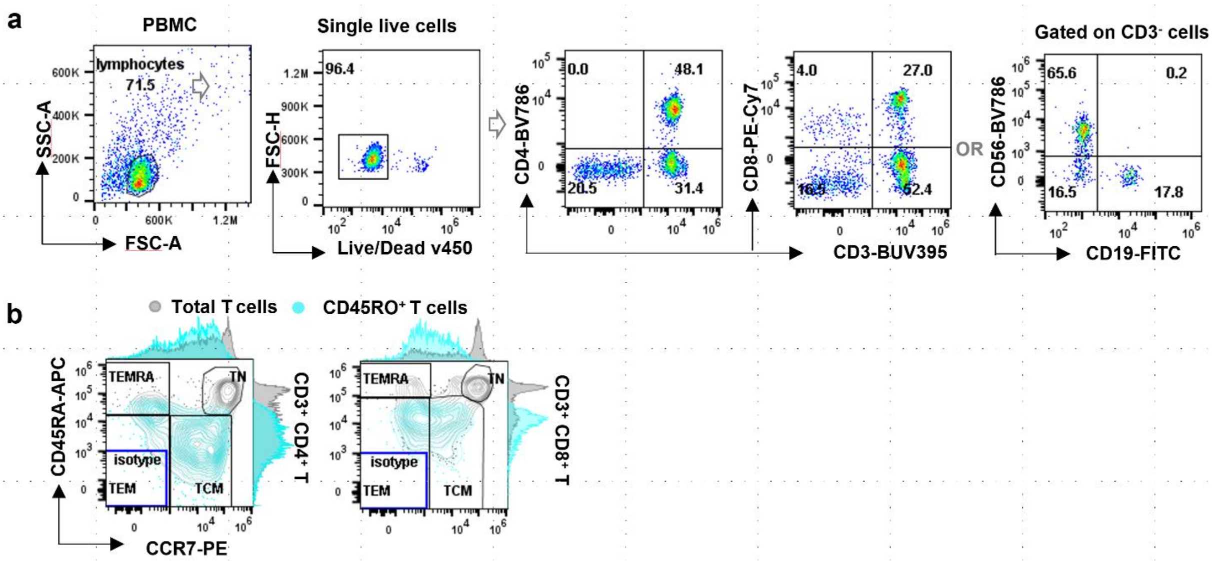

Figure S1 | Gating methods to identify main lymphocyte populations and T-cell subsets. **a**) Gating strategies to identify $CD3^+$ $CD4^+$ T, $CD3^+$ $CD8^+$ T, NK and B cells. **b**) Characterization of T cell naïve/memory lineages including T_N, T_{CM}, T_{EM}, and T_{EMRA} based on the expression of canonical markers CCR7/CD45RA/CD45RO. The blue line represents CCR7/CD45RA isotype gate.

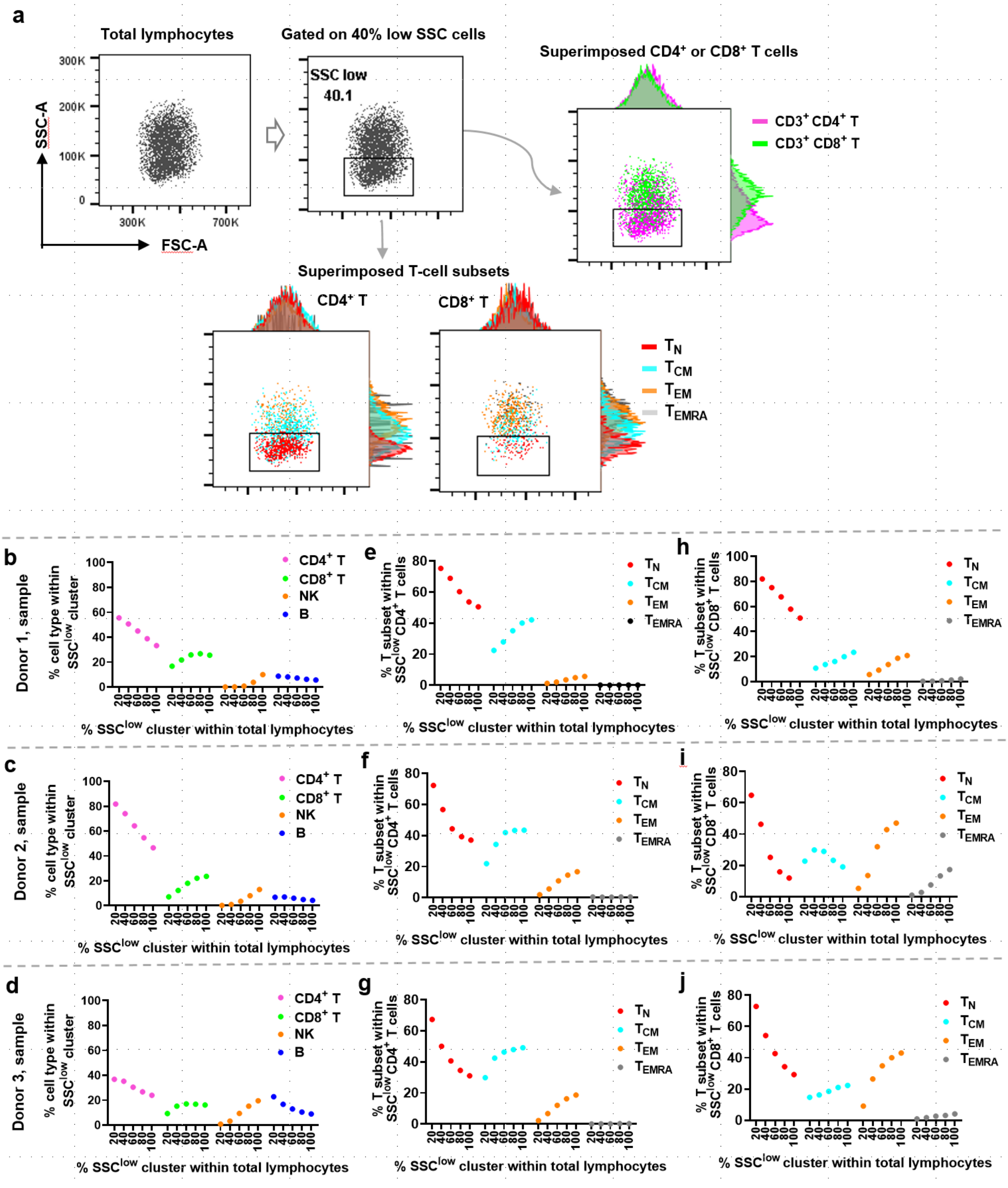

Figure S2 | Gate selection based on SSC intensity and composition change of lymphocyte subpopulations. a) Representative gating where 40% of the total lymphocytes with low SSC intensity were selected. The total $CD4^+$ T, $CD8^+$ T, or their refined cell subsets were superimposed onto the gating plot to show their scattering distribution within and outside the gate. **b**–**j**) Percentages of $CD3^+$ $CD4^+$ T, $CD3^+$ $CD8^+$ T, NK, and B cells (**b**–**d**), or T cell naïve/memory subsets in $CD3^+$ $CD4^+$ T (**e**–**g**) and $CD3^+$ $CD8^+$ T cells (**h**–**j**) among the gated SSC^{low} cluster (n = 3).

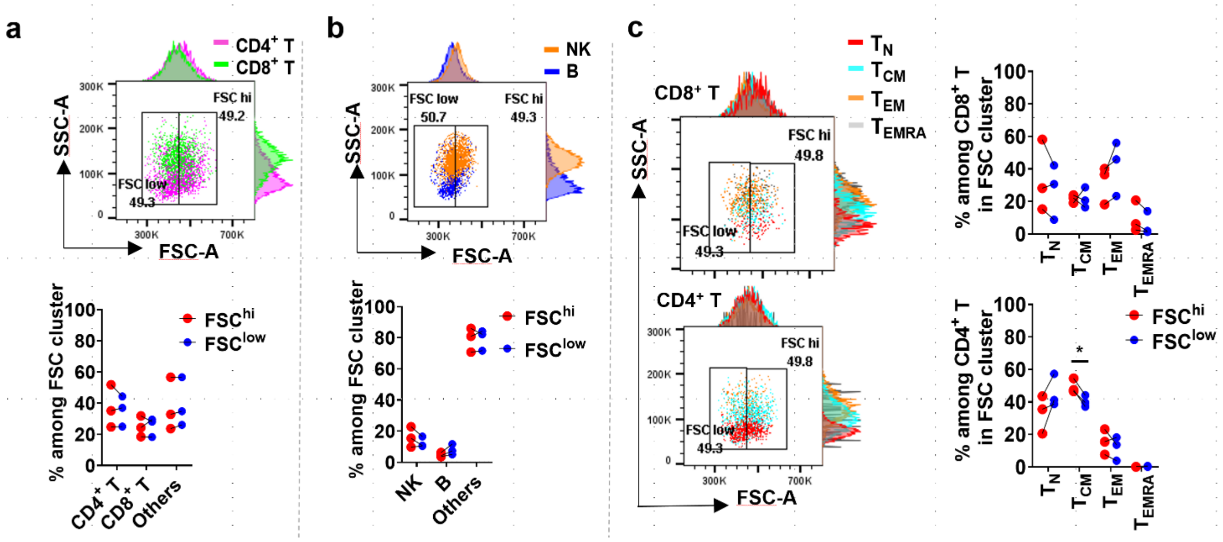

Figure S3 | FSC is less efficient in segregating lymphocyte subpopulations. a–**c**) PBMCs were pre-stained with lineage (**a**,**b**) or differentiation-specific markers (**c**). A gating method based on the FSC intensity was used to divide total lymphocytes into FSC^{hi} and FSC^{low} clusters as illustrated in the dot plots, correspondingly. The percentage of lymphocyte subpopulations within the FSC^{hi} or FSC^{low} cluster was summarized (n = 3). *p < .05, **p < .01, paired two-tailed t-test.

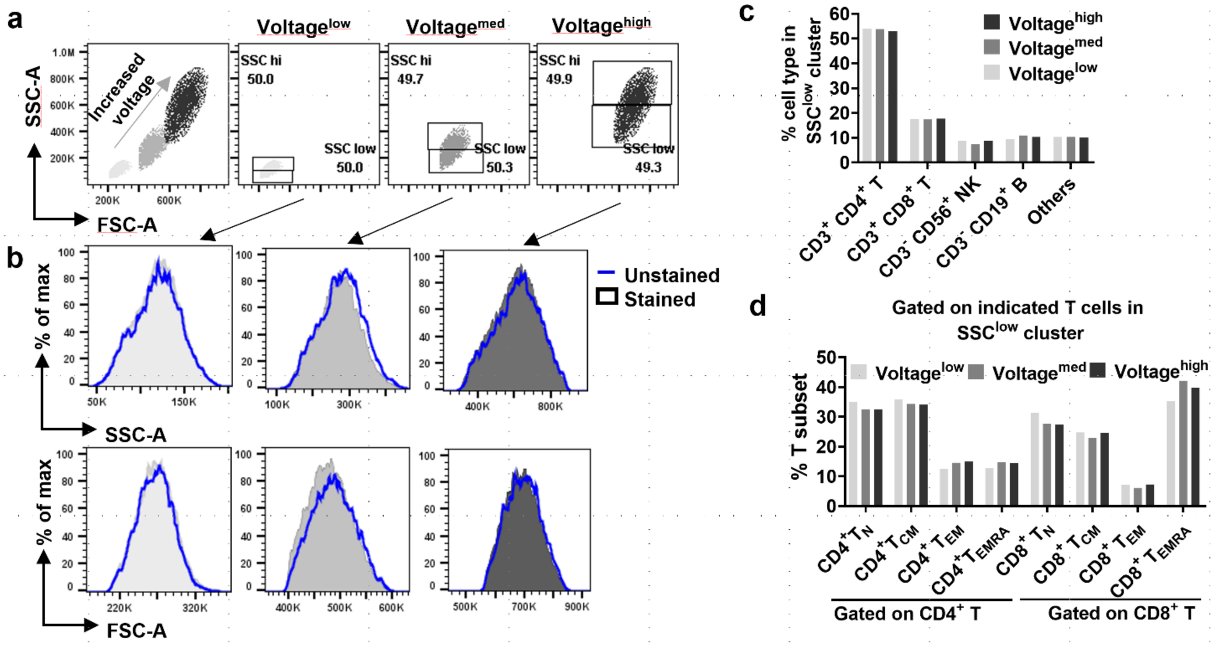

Figure S4 | SSC for segregating lymphocyte subpopulations. a) Pre-stained PBMC was analyzed under different voltages for FSC and SSC data acquisition. **b**) Control cells were unstained but subjected to the same sample processing steps. Shown is the scattering profile of total lymphocytes with or without antibody staining. **c**,**d**) The percentage of $CD3^+$ $CD4^+$ T, $CD3^+$ $CD8^+$ T, NK, and B cells in gated SSC^{low} cluster (**c**), as well as the naive/memory T-cell subsets in $CD4^+$ T and $CD8^+$ T lymphocyte among the gated SSC^{low} cell population (**d**) were compared under different voltages during data acquisition. Two-way ANOVA test was performed and no significant difference (p = 0.999) was observed between the cell composition as recovered from the sorting under different voltages.

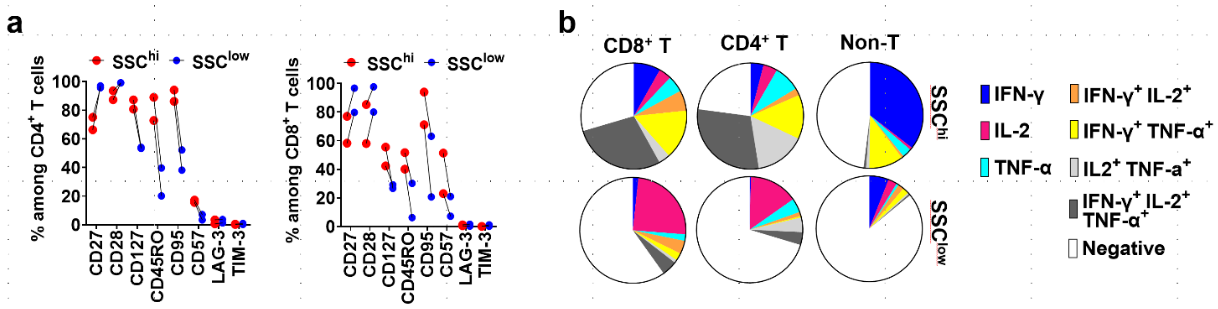

Figure S5 | In-depth characterization of phenotypes and functionality of enriched younger T-cell subsets. a) Expression profiles of selected phenotyping markers in sorted SSC^{high} and SSC^{low} cells. Each dot represents data of one donor sample (n = 2). **b**) Sorted SSC^{high} and SSC^{low} cells were stimulated with PMA plus ionomycin for 4 hours before intracellular staining. Shown are the multifunctional cytokine expression of CD3^{+} CD8^{+} T, CD3^{+} CD8^{-} T (CD4^{+} T) cells, and CD3^{-} (Non-T) cells within sorted SSC^{high} or SSC^{low} cells (n = 2).

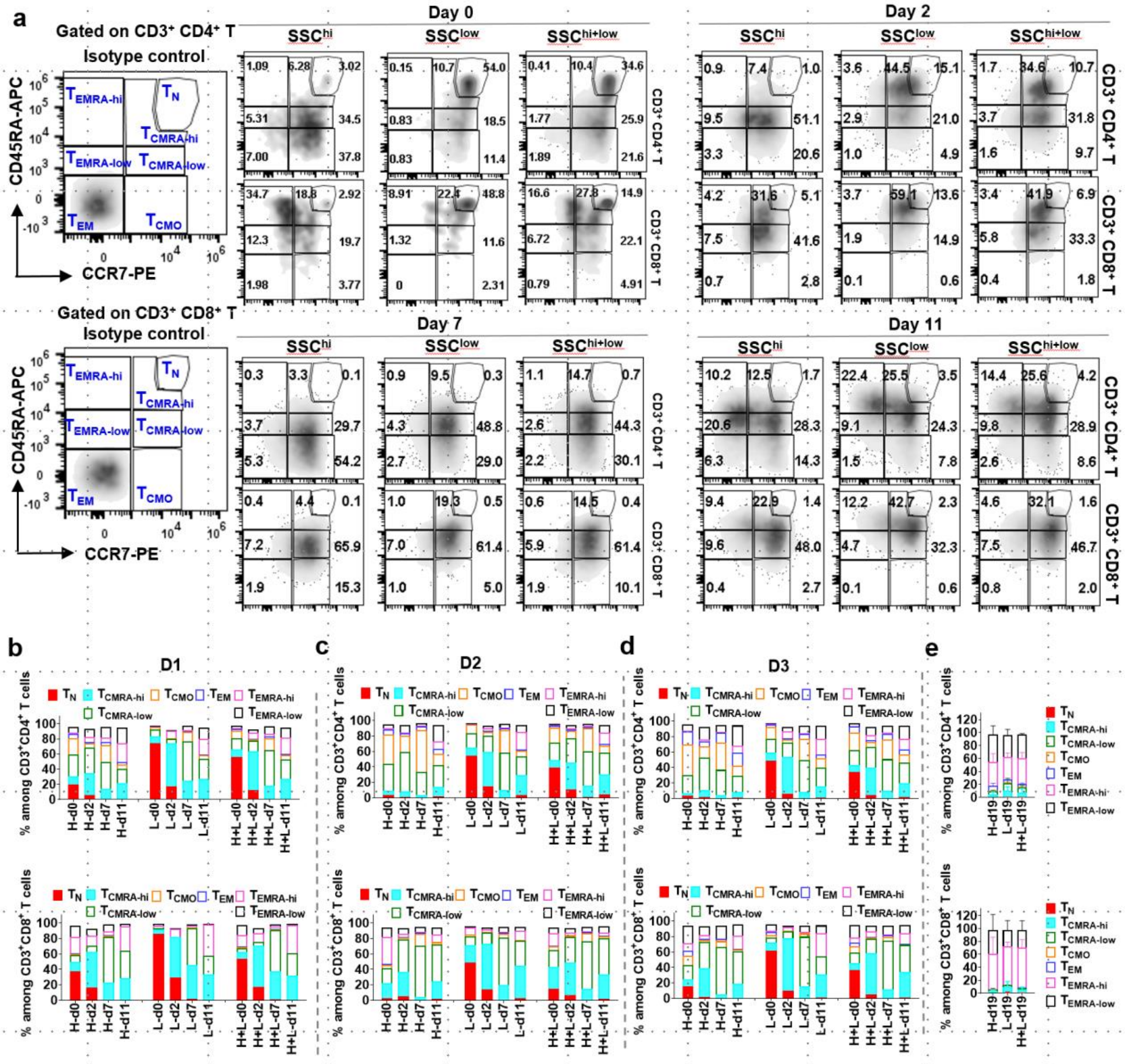

Figure S6 | Dynamic phenotypic change of individual lymphocyte samples sorted by SSC.

a) Gating strategy for in-depth analysis of compositional change of naïve/memory T cells over expansion course. **b**–**d**) Composition change of naive/memory T-cell subsets in $CD3^+$ $CD4^+$ T or $CD3^+$ $CD8^+$ T cells for each donor sample along the expansion procedure. **e**) T cell naïve/memory composition at day 19 post-expansion. Error bars indicate mean ± SEM (n = 2).

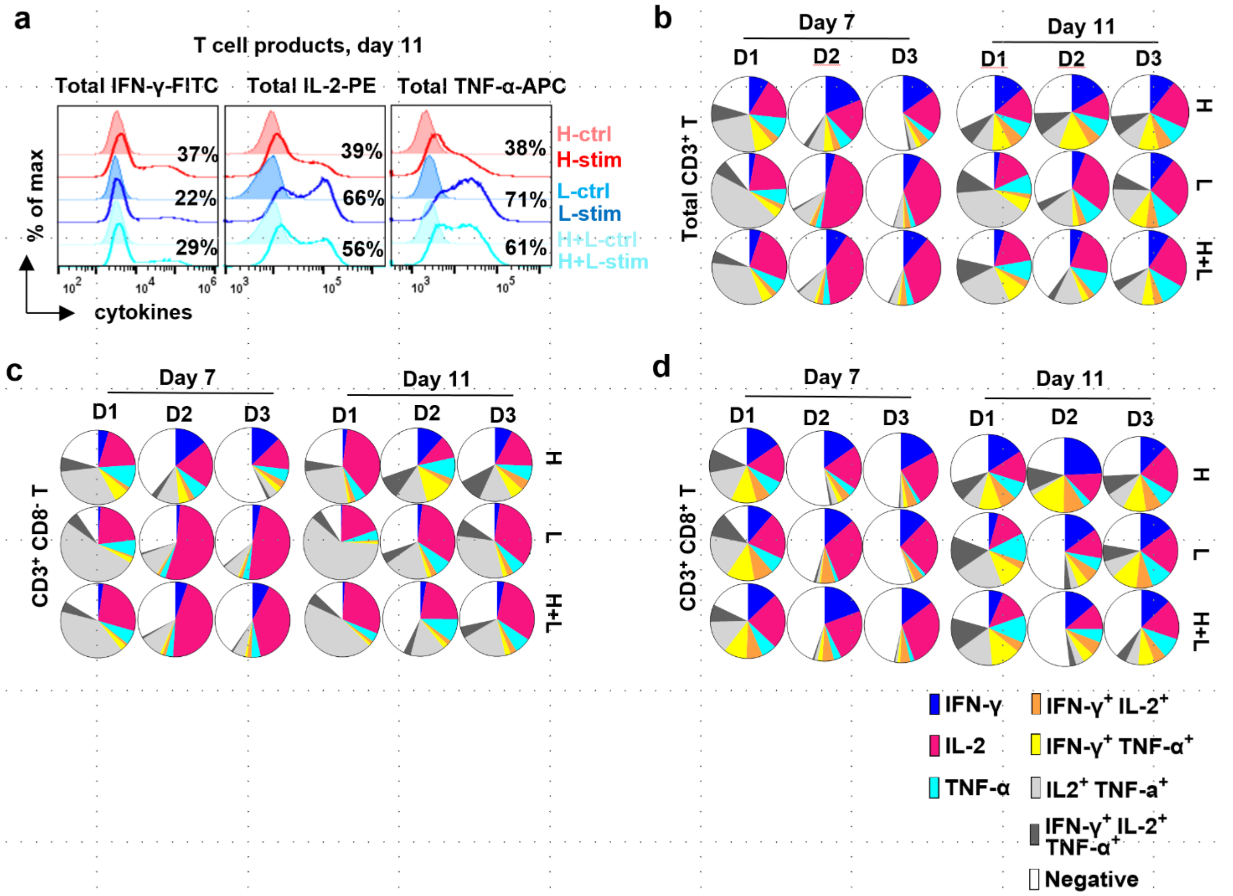

Figure S7 | In-depth functional analysis of expanded T cells. a) Representative histogram plot of IFN-γ/IL-2/TNF-α expression of expanded T cells (day 11) derived from cell groups initially with different SSC intensity. The cells were stimulated with 50 ng/ml PMA and 1 µg/ml ionomycin ("stim" group) or kept unstimulated ("ctrl" group) for additional 2.5 hours before cytokine production detection. **b–d**) Cytokine secretion profile of expanded total $CD3^+$ T (**b**), $CD3^+$ $CD8^-$ T (**c**), and $CD3^+$ $CD8^+$ T cells (**d**). "H" and "L" indicate "SSC^{high}" and "SSC^{low}", respectively.

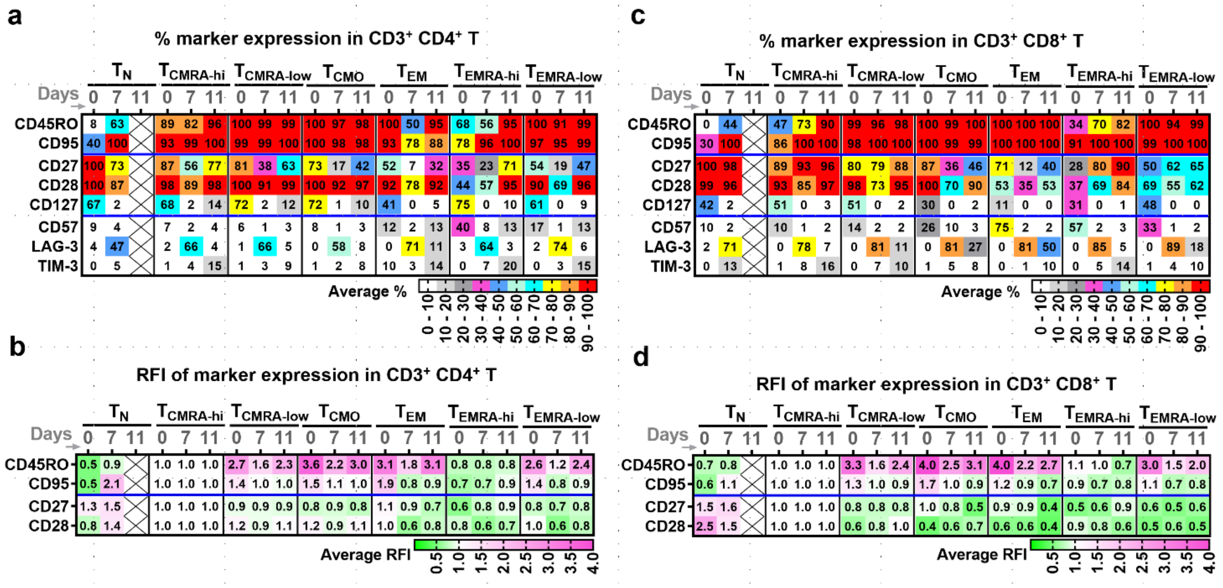

Figure S8 | In-depth phenotypic analysis of expanded CD4⁺ T and CD8⁺ T cells. a–d) Longitudinal assessment of phenotypic change of $T_{CMRA\text{-}hi}$ compared to other T cell subsets. Average expression and abundance per cell of selected phenotypic markers within $CD4^+$ T (**a**,**b**) or $CD8^+$ T subsets (**c**,**d**) (n = 3). The relative fluorescence intensity (RFI) was reported after normalization to that of $T_{CMRA\text{-}hi}$ of the same time points respectively (**b**,**d**). The cross label indicates omitted data due to low cell percentages for marker expression identification or insufficient counts for MFI calculation.

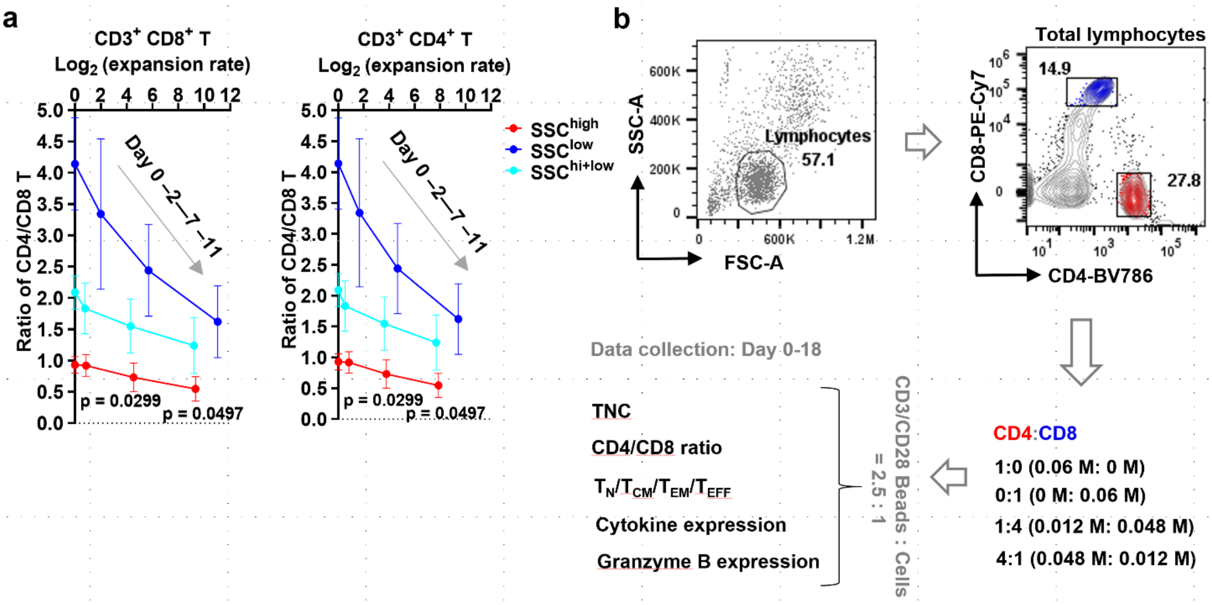

Figure S9 | Study of CD4$^+$ T help in regulating CD8$^+$ T proliferation and function. a) Synchronized T cell expansion potency with the ratio of CD4/CD8 T cells starting from cell materials of different SSC intensity. Data are mean ± S.E.M (n = 3). **b**) CD4$^+$ cells and CD8$^+$ cells were sorted and validated to contain more than 96% of CD3+ CD4+ T and CD3$^+$ CD8$^+$ T cells, respectively. CD4$^+$ cells and CD8$^+$ cells were reconstituted at indicated ratio and co-expanded by CD3/CD28 engagement.

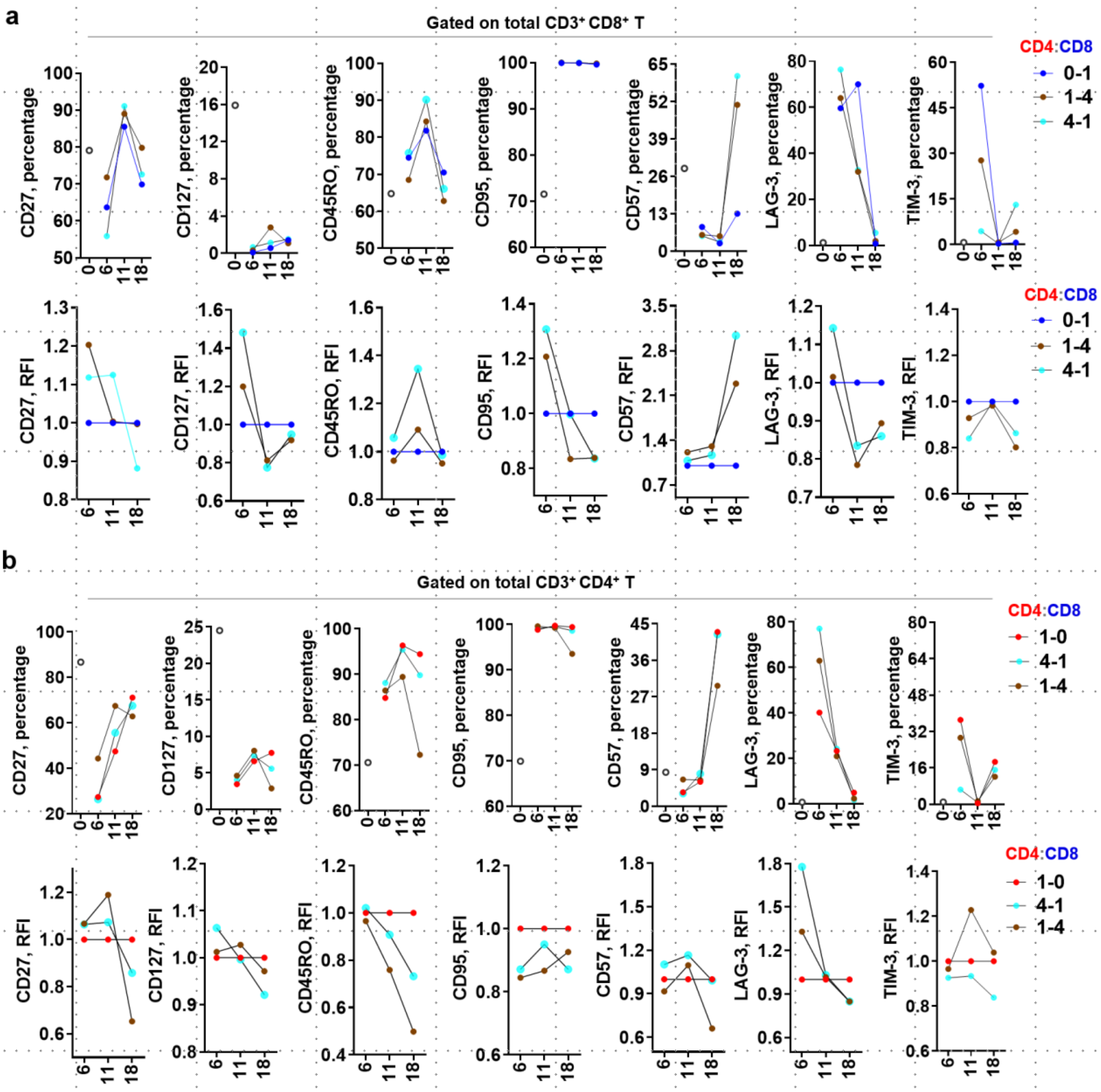

Figure S10 | Expression profiles of selected phenotypic markers in co-expanded T cells. **a**,**b**) Dynamic expression of a panel of phenotypic markers in CD3$^+$ CD4$^+$ T (**a**) or CD3$^+$ CD8$^+$ T cells (**b**) at day 6, 11, and 18 after CD4-CD8 T co-expansion. The relative fluorescence intensity (RFI) was calculated by normalizing the medium fluorescence intensity (MFI) of each marker under CD4-CD8 co-culture conditions to that of CD4$^+$ T cells or CD8$^+$ T cells without co-culture at the indicated time points.

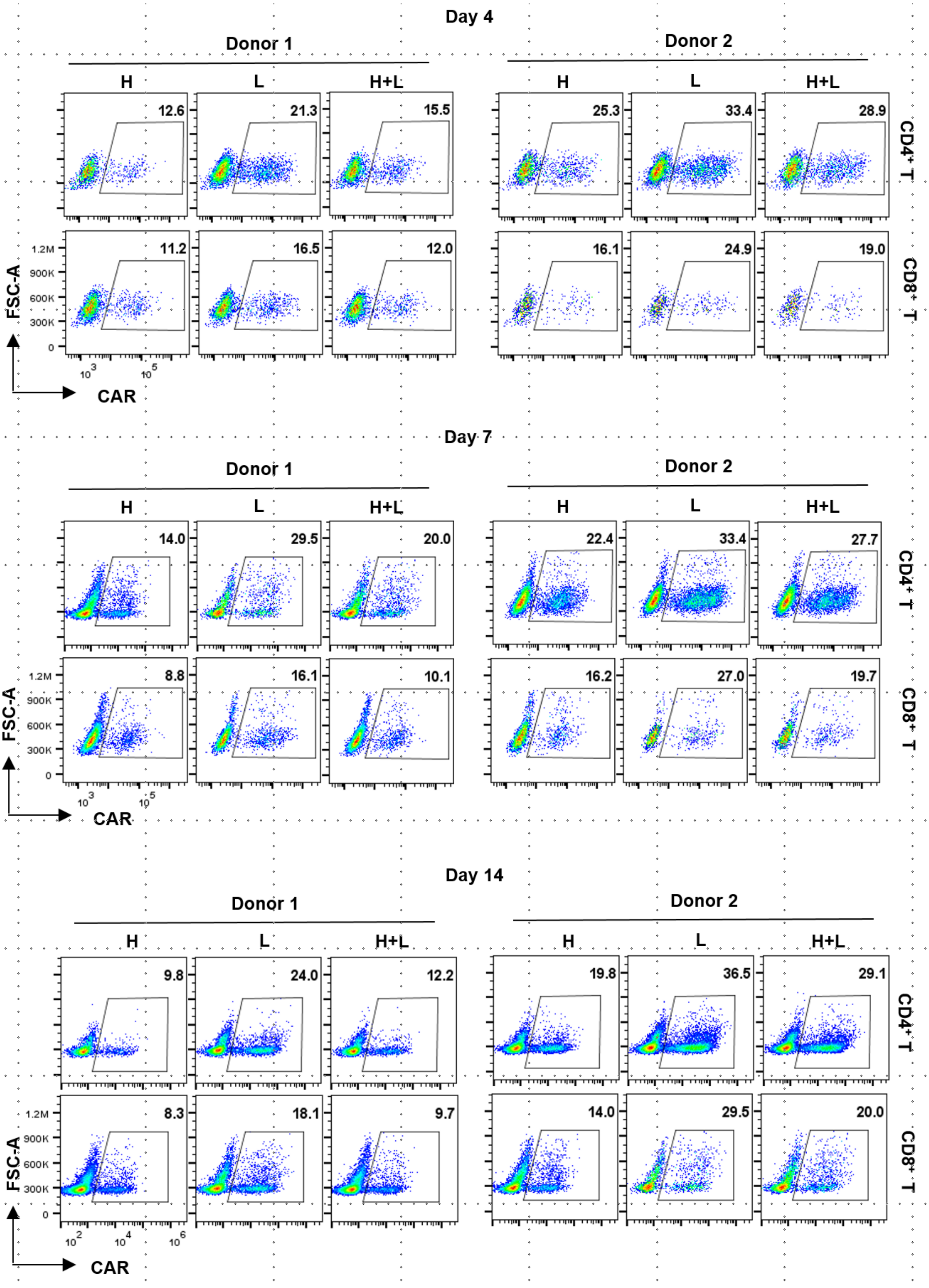

Figure S11 | Representative CAR expression over time.

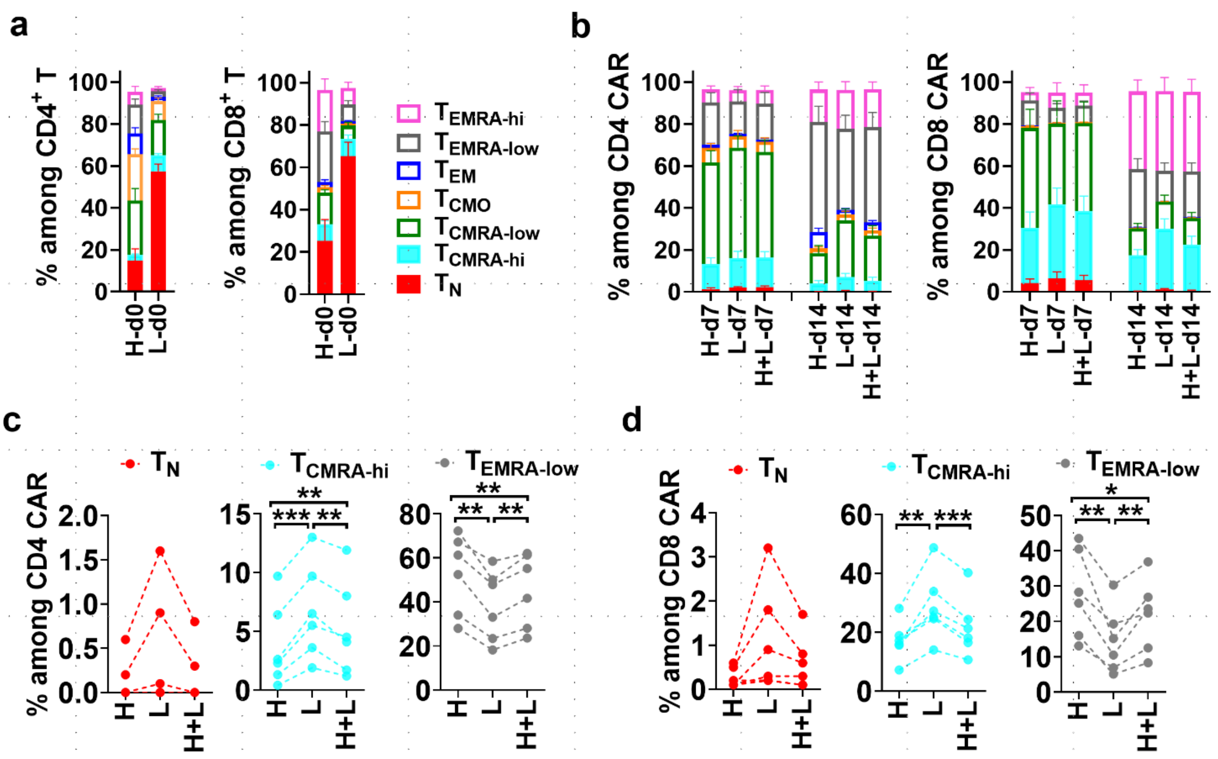

Figure S12 | T-cell composition before and after CAR-T production. a,**b**) Longitudinal assessment of T-cell differentiation subsets before (**a**) and after CAR-T generation (**b**) over the expansion course. Shown are mean ± SEM (n = 6). **c**,**d**) Percentage of T_N, $T_{CMRA\text{-}hi}$, and $T_{EMRA\text{-}low}$ among CD4$^+$ CAR-T or CD8$^+$ CAR-T cells at day 14 after expansion (n = 6). Data points from the same donor sample were connected respectively. Data are mean ± S.E.M (n = 6) (**a**,**b**). *$p < .05$, **$p < .01$, ***$p < .001$, paired two-tailed *t*-test (n = 6) (**c**,**d**).

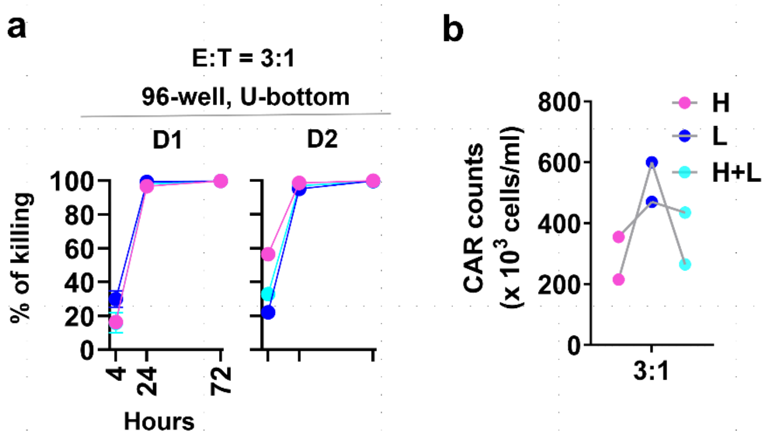

Figure S13 | Comparable target-killing activity of different CAR-T cells at high E-T ratio.

a,**b**) CAR-T and NALM6.Luc cells were co-cultured in 96-well round-bottom culture plates at a ratio of 3:1. Killing efficiency was calculated according to the reduction of luciferase intensity in the presence of CAR-T compared to NALM6.Luc cells alone (**a**). CAR-T numbers on day 3 after co-culture were manually counted (**b**).

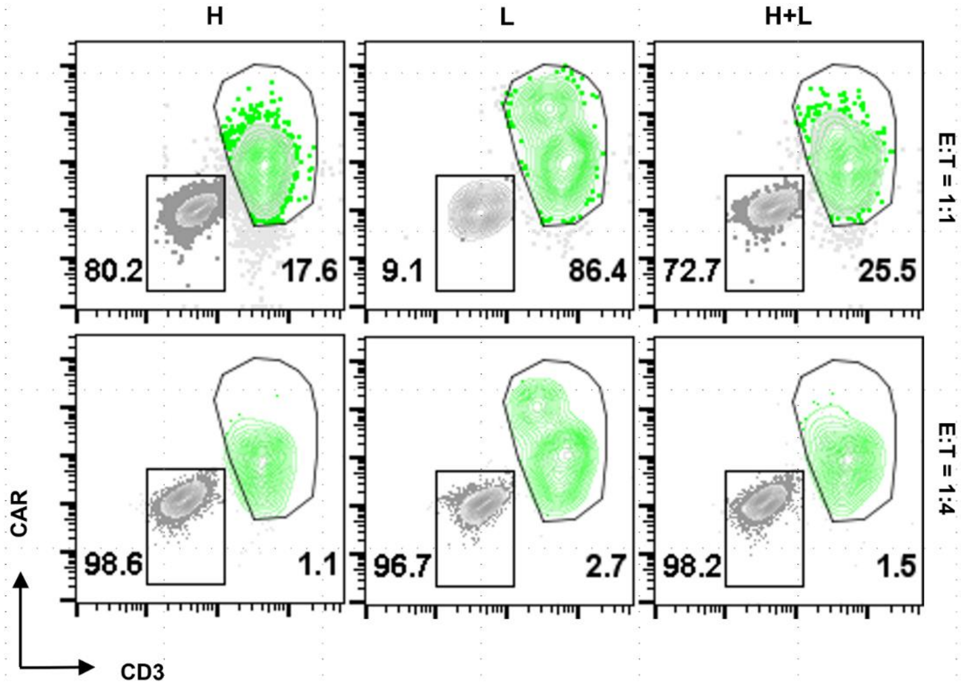

Figure S14 | Distinguishable target-killing activity of different CART19 cells at low E-T ratio. CAR-T and NALM6.Luc cells were co-cultured in 24-well flat-bottom culture plates at low E:T ratio as indicated. Representative scatter plots showing the percentage of remaining CAR-T and NALM6.Luc cells at day 3 after co-culture.

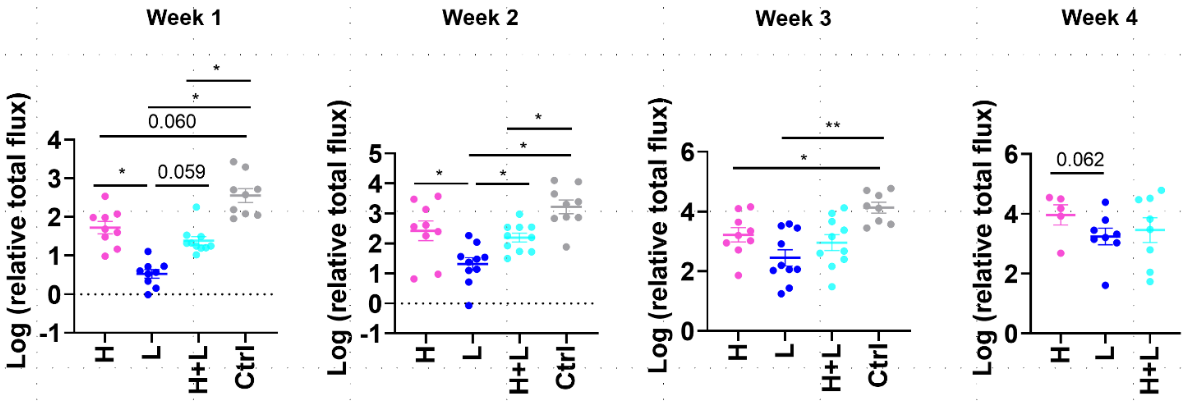

Figure S15 | Tumor growth over time. Quantification of tumor burden from week 0 to week 4 by measuring total photon flux per second and shown were data of each mouse at individual time points. Data are mean ± S.E.M, unpaired two-tailed t-test. *$p < .05$, **$p < .01$. Exact *p*-values for moderate but non-significant trends were also indicated.

Sidak's multiple comparisons test (week 1-3)					
	Mean Diff.	95.00% CI of diff.	Significant?	Summary	Adjusted P Value
H *versus* L					
Week 1	1.197	0.6710 to 1.723	Yes	****	<0.0001
Week 2	1.107	0.05422 to 2.161	Yes	*	0.0382
Week 3	0.7711	-0.1848 to 1.727	No	ns	0.136
L *versus* H+L					
Week 1	-0.8583	-1.267 to -0.4496	Yes	***	0.0001
Week 2	-0.8785	-1.560 to -0.1970	Yes	*	0.0101
Week 3	-0.5091	-1.510 to 0.4919	No	ns	0.4832

Sidak's multiple comparisons test (week 1-4)					
	Mean Diff.	95.00% CI of diff.	Significant?	Summary	Adjusted P Value
H *versus* L					
Week 1	1.197	0.6429 to 1.752	Yes	****	<0.0001
Week 2	1.107	-0.002385 to 2.217	No	ns	0.0506
Week 3	0.7711	-0.2341 to 1.776	No	ns	0.1771
Week 4	0.7212	-0.6475 to 2.090	No	ns	0.4439
L *versus* H+L					
Week 1	-0.8583	-1.288 to -0.4284	Yes	***	0.0001
Week 2	-0.8785	-1.596 to -0.1615	Yes	*	0.0135
Week 3	-0.5091	-1.561 to 0.5430	No	ns	0.5852
Week 4	-0.2147	-1.663 to 1.233	No	ns	0.9886

Figure S16 | Post-hoc test following ANOVA analysis.

For the comparison of tumor growth over time between different mice groups. Two-way ANOVA analysis with Sidak's multiple comparisons test was performed. Shown were the results of post-hoc test along with the ANOVA test in Figure 8c. $^{*}p < .05$, $^{**}p < .01$, $^{***}p < .001$, $^{****}p < .0001$.

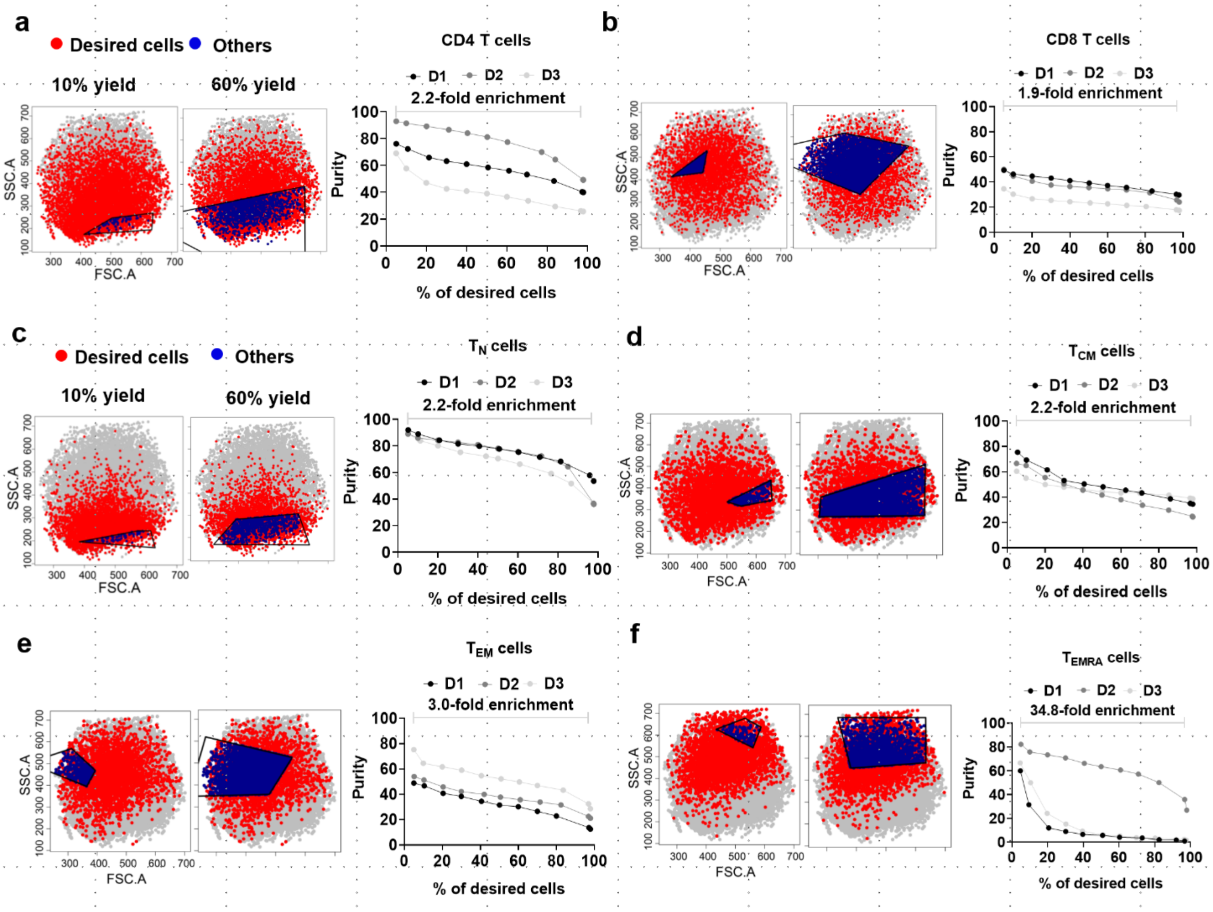

Figure S17 | Computational gate search for T-cell subpopulation selection.
The raw values corresponding to FSC-A and SSC-A parameters were exported and reanalyzed by the GateID algorithm to select the desired T-cell subpopulations. Shown are the enrichment efficiency of $CD3^+$ $CD4^+$ T (**a**), $CD3^+$ $CD8^+$ T (**b**), total T_N (**c**), T_{CM} (**d**), T_{EM} (**e**), and T_{EMRA} subsets (**f**) under automatically adjusted gating threshold. For each cell population, shown are two representative scatter plots corresponding to 10% and 60% yields of desired cells, and the correlation plots between purity and yield. An average enrichment fold increase from ~100% yield to ~5% yield (n = 3) is indicated. The percentage of desired cells (yield) is calculated as the number of desired cells within the gating to that of total desired cells among the whole cell population. The purity indicates the proportion of desired cells to that of total cell numbers within each gate.
